# Plasma Engineering of Basal Sulfur Sites on MoS_2_@Ni_3_S_2_ Nanorods for the Alkaline Hydrogen Evolution Reaction

**DOI:** 10.1002/advs.202104774

**Published:** 2021-12-22

**Authors:** Xin Tong, Yun Li, Qingdong Ruan, Ning Pang, Yang Zhou, Dajun Wu, Dayuan Xiong, Shaohui Xu, Lianwei Wang, Paul K. Chu

**Affiliations:** ^1^ Key Laboratory of Polar Materials and Devices (MOE) Department of Electronics East China Normal University Shanghai 200241 P. R. China; ^2^ Jiangsu Laboratory of Advanced Functional Materials School of Electronic and Information Engineering Changshu Institute of Technology Changshu 215500 P. R. China; ^3^ Department of Physics Department of Materials Science and Engineering and Department of Biomedical Engineering City University of Hong Kong Tat Chee Avenue Kowloon Hong Kong China; ^4^ School of Physics and Electronic Engineering Hanshan Normal University Chaozhou 521041 P. R. China

**Keywords:** active sites, hydrogen evolution reaction, interface engineering, plasma doping, 2D nanomaterials

## Abstract

Inexpensive and efficient catalysts are crucial to industrial adoption of the electrochemical hydrogen evolution reaction (HER) to produce hydrogen. Although two‐dimensional (2D) MoS_2_ materials have large specific surface areas, the catalytic efficiency is normally low. In this work, Ag and other dopants are plasma‐implanted into MoS_2_ to tailor the surface and interface to enhance the HER activity. The HER activty increases initially and then decreases with increasing dopant concentrations and implantation of Ag is observed to produce better results than Ti, Zr, Cr, N, and C. At a current density of 400 mA cm^−2^, the overpotential of Ag500‐MoS_2_@Ni_3_S_2_/NF is 150 mV and the Tafel slope is 41.7 mV dec^−1^. First‐principles calculation and experimental results reveal that Ag has higher hydrogen adsorption activity than the other dopants and the recovered S sites on the basal plane caused by plasma doping facilitate water splitting. In the two‐electrode overall water splitting system with Ag500‐MoS_2_@Ni_3_S_2_/NF, a small cell voltage of 1.47 V yields 10 mA cm^−2^ and very little degradation is observed after operation for 70 hours. The results reveal a flexible and controllable strategy to optimize the surface and interface of MoS_2_ boding well for hydrogen production by commercial water splitting.

## Graphical abstract

The injection of Ag plasma into MoS_2_ can better adjust the surface interface function to enhance the activity of hydrogen evolution reaction. These are mainly derived from injecting an appropriate amount of Ag into MoS_2_, which not only maintains the large surface area structure, but also activates the basal plane S atoms connected to the Ag impurities.

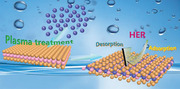

## 1 Introduction

Development of the modern society demands green energy and sustainable environment^[^ ^1^ ^]^ and in order to alleviate the shortage of fossil fuel and greenhouse gas emission, hydrogen production by water electrolysis or water splitting is one of the important strategies.^[^ ^2^ ^]^ In the hydrogen evolution reaction (HER) which is one of the two reactions in water splitting, the best catalysts so far are Pt‐based but they suffer from the high price and natural scarcity and therefore, more efficient and economical electrocatalysts are desirable.^[^ ^3^, ^4^ ^]^ Some transition metal compounds such as Co‐based, Ni‐based, Fe‐based, and Mo‐based materials have received attention as catalysts because of the low price, abundant reserve, and special physical properties.^[^ ^5^, ^6^, ^7^, ^8^, ^9^, ^10^ ^]^ For example, Mo‐based catalysts such as MoSe_2_ nanosheets, MoP/carbon nanotube hybrids, Ni/MoC heteronanoparticles, MoNi_4_/MoO_3−_ *_x_* nanorod arrays, MoS_2_‐based catalysts, and so on have been proposed.^[^ ^11^, ^12^, ^13^, ^14^, ^15^ ^]^ Among them, MoS_2_ materials have great potential as substitutes for Pt‐based catalysts in HER due to the unique 2D layer structure and chemical properties.^[^ ^16^ ^]^ However, it has been shown that the active sites on pure MoS_2_ are mainly the S sites at the edge, but the S sites on the basal plane are inactive^[^ ^17^ ^]^ and activation of the S sites on the basal plane of MoS_2_ is still challenging.^[^ ^18^ ^]^

The modification strategies of MoS_2_ follow the following main directions.^[^ ^19^ ^]^ First, construction of new active sites or activation of S sites on the basal plane can be accomplished by doping MoS_2_.^[^ ^20^ ^]^ For example, Tian et al. have reported a MoS_2_ nanofoam catalyst containing selenium on the surface and cobalt in the inner layer showing good HER activity.^[^ ^21^ ^]^ Qi et al. have proposed that introducing Pd atoms to the basal plane of defect‐rich MoS_2_ produces Pt‐like HER properties such as low onset overpotential, small Tafel slope, and HER durability.^[^ ^22^ ^]^ Second, heterostructures of MoS_2_ can be produced in combination with other suitable materials to activate the S sites on the surface.^[^ ^23^ ^]^ For instance, Zhao et al. have synthetized 3D graphene aerogel supported layered MoS_2_ nanosheets by self‐assembly with high catalytic activity and durability in HER.^[^ ^24^ ^]^ Third, by adjusting the lattice spacing and interface of MoS_2_,^[^ ^25^ ^]^ Wei et al. have demonstrated a technique to activate the monolayer MoS_2_ basal plane by introducing domain boundaries.^[^ ^26^ ^]^ Although modification of the surface of MoS_2_ to activate the S sites can indeed improve the activity in HER, there have been few comprehensive studies on the mechanism and effects of the type and amount of dopants to attain the optimal performance.^[^ ^27^ ^]^ In fact, there has been little work on the use of plasmas to achieve precise control of the active sites on catalysts.^[^ ^9^ ^]^ Since plasma processes are widely used in the industry, especially microelectronics processing, it is important to study the effects of different dopants and plasma parameters such as fluence on the catalytic activity of HER catalysts.^[^ ^28^, ^29^ ^]^

Herein, Ag, Zr, Ti, Cr, C, and N are plasma‐implanted into MoS_2_@Ni_3_S_2_ nanorods with different fluences to investigate the effects on the catalytic sites. Our results reveal that Ag ion implantation with the proper fluence leads to the best HER activity. The experimental and theoretical results show that the right amount of implanted Ag in MoS_2_ not only maintains the 2D structure with a large surface area, but also introduces a large number of active centers to reduce the hydrogen adsorption energy. The active sites for HER is the S sites on the MoS_2_ basal surface directly connected to the doped atoms. The strategy involving structural engineering and plasma processing renders MoS_2_@Ni_3_S_2_ an excellent catalyst for HER, the concept and materials provide insights into the development of high‐efficiency water splitting catalysts.

## 2 Results and Discussion

### 2.1 Materials and Characterization

**Figure 1:**
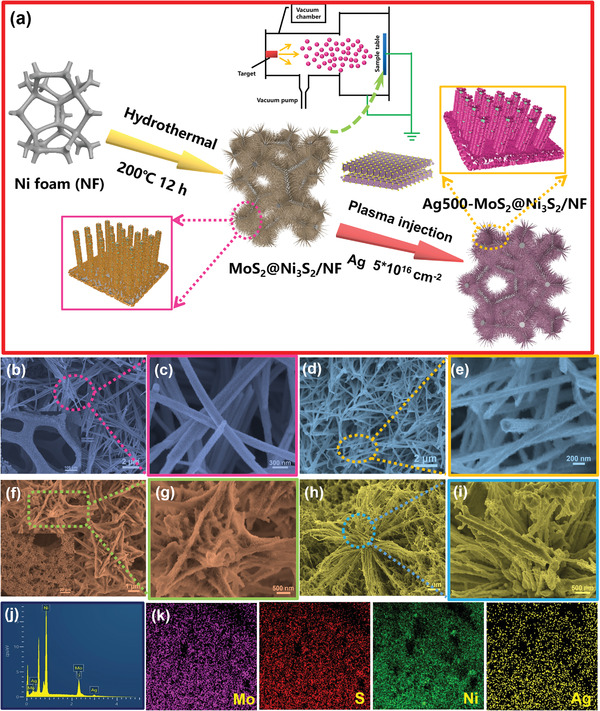
a) Schematic illustration of the preparation of Ag‐doped MoS_2_@Ni_3_S_2_/NF nanorods by plasma doping; SEM images of b,c) MoS_2_@Ni_3_S_2_/NF, d,e) Ag250‐MoS_2_@Ni_3_S_2_/NF, f,g) Ag500‐MoS_2_@Ni_3_S_2_/NF, and h,i) Ag1000‐MoS_2_@Ni_3_S_2_/NF; j) EDS spectrum of Ag500‐MoS_2_@Ni_3_S_2_/NF; k) EDS elemental maps of Ag500‐MoS_2_@Ni_3_S_2_/NF.

**Figure** **1**a illustrates the process in which the MoS_2_@Ni_3_S_2_ nanorods are prepared on nickel foam (NF) hydrothermally and subsequently modified by plasmas. In the first hydrothermal step, uniform and elongated MoS_2_@Ni_3_S_2_ nanorods are produced vertically on the conducting NF framework. The SEM images in Figure 1b,c disclose that the pristine MoS_2_@Ni_3_S_2_ has a nanorod structure with a length of 2 µm and width of 150 nm. During plasma implantation, different elements are implanted into the MoS_2_@Ni_3_S_2_ nanorods with different fluences. As plasma implantation proceeds, the fluence increases and the nanorods are interconnected to form a nanoflower‐like or nanonet‐like structure. The SEM images of the modified MoS_2_@Ni_3_S_2_ nanorods implanted with different amounts of Ag are depicted in Figure 1d–i. The morphology of the lightly doped MoS_2_@Ni_3_S_2_ is basically unchanged (Figure 1d,e) but for higher fluences, the nanorods aggregate to form a flower‐like structure (Figure 1f,g) and finally, the nanorods agglomerate to form a network (Figure 1h,i). This is mainly because the Ag plasma is accelerated by the 25 kV acceleration grid to obtain higher energy and accelerate to the sample surface on the grounded sample stage. Due to its relatively large energy, the morphology of the sample will be greatly changed after a large dose of incorporation to relieve stress. For comparison, the morphology and size of the MoS_2_@Ni_3_S_2_/NF nanorods doped with different elements and concentrations are displayed in Figures S2–S6, Supporting Information. The morphological changes are basically consistent with that observed from the Ag‐implanted samples. As shown in Figure 1j,k, Mo, S, Ag, and Ni are distributed uniformly on the surface of Ag500‐MoS_2_@Ni_3_S_2_.

**Figure 2:**
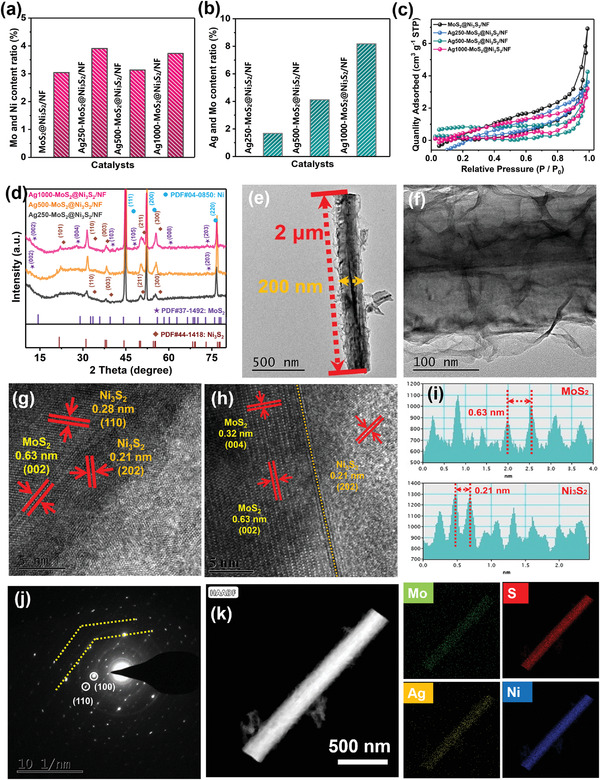
a,b) Mo/Ni and Ag/Mo atomic ratios of Ag‐doped MoS_2_@Ni_3_S_2_/NF determined by ICP; c) Nitrogen adsorption–desorption isotherms of MoS_2_@Ni_3_S_2_/NF with different Ag concentrations; d) XRD patterns of Ag‐doped MoS_2_@Ni_3_S_2_/NF; e,f) TEM images of Ag500‐MoS_2_@Ni_3_S_2_/NF; g,h) HRTEM images; i) Lattice spacing distribution; j) SEAD pattern; k) TEM/EDS elemental maps.

The atomic ratios of the metallic elements in the catalysts are determined by inductively coupled plasma atomic emission spectroscopy (ICP‐AES). The Mo/Ni ratios in MoS_2_@Ni_3_S_2_/NF doped with different amounts of Ag are 3–4% and the amounts of MoS_2_ in the materials are uniform and stable (**Figure** **2**a). The ratio of Ag to Mo is less than 10% in the near surface as a result of the Gaussian distribution of ion implantation and so the implanted Ag mainly exists as dopants on the MoS_2_ surface (Figure 2b). The surface area is one of the important parameters to evaluate the electrocatalytic ability. As shown in Figure 2c, the specific surface areas of the pristine MoS_2_@Ni_3_S_2_/NF, Ag250‐MoS_2_@Ni_3_S_2_/NF, Ag500‐MoS_2_@Ni_3_S_2_/NF, and Ag1000‐MoS_2_@Ni_3_S_2_/NF are 2.57, 2.34, 1.73, and 2.50 m^2^ g^−1^, respectively. By means of the Barrett–Joyner–Halena adsorption model, the pore sizes of the samples with different amounts of implanted Ag are 4.44, 2.52, 1.19, and 1.76 nm, indicating that MoS_2_@Ni_3_S_2_/NF and Ag250‐MoS_2_@Ni_3_S_2_/NF have mesoporous characteristics and Ag500‐MoS_2_@Ni_3_S_2_/NF and Ag1000‐MoS_2_@Ni_3_S_2_/NF exhibit microporous characteristics (Figure S7, Supporting Information). Catalysts with more micropores have more surface areas which may lead to a larger number of active sites and better contact between the active sites and substrate.^[^ ^30^ ^]^ Therefore, although the specific surface area is similar, Ag500‐MoS_2_@Ni_3_S_2_/NF has more micropores suggesting more active sites and perhaps higher catalytic activity.^[^ ^31^ ^]^

Figure 2d shows the three typical diffraction peaks at 44.5, 52.1, and 76.7° corresponding to the (111), (200), and (220) planes of the Ni substrate (PDF#04‐0850).^[^ ^32^ ^]^ The diffraction peaks marked by squares can be indexed to the (101), (110), (003), (211), and (300) planes of the Ni_3_S_2_ nanorods (PDF#44‐1418)^[^ ^33^ ^]^ and those at 12.4, 27.8, 38.9, 47.9, 58.8, and 71.4° marked by pentagons are the (002), (004), (103), (105), (008), and (203) planes of the MoS_2_ nanosheets (PDF#37‐1492).^[^ ^34^ ^]^ The reason for the small diffraction peaks of MoS_2_ is mainly because the content of MoS_2_ is very low compared to Ni_3_S_2_, and the depth of X‐ray diffraction (XRD) measurement is also very deep to several microns. Therefore, the diffraction peak of MoS_2_ is not clear enough on a strong Ni and Ni_3_S_2_ substrate. However, the still visible diffraction peaks from MoS_2_ shift to smaller angles in general because the atomic radius of Ag is larger than that of Mo thus increasing the interplanar spacing. Moreover, the XRD diffraction peaks of Ni_3_S_2_ shifts to the high angle direction with the increase of Ag doping, mainly due to the extrusion caused by the high‐energy plasma injection process, which makes the lattice spacing smaller. The XRD patterns of the other plasma‐doped catalysts in Figure S8, Supporting Information, show that the diffraction peaks from MoS_2_ shift to larger angles because the atomic radii of Zr, Ti, Cr, C, and N are smaller than those of Mo and S, so that the unit cell parameters of MoS_2_ are slightly reduced.

The morphology and lattice parameters of Ag500‐MoS_2_@Ni_3_S_2_/NF are studied by transmission electron microscopy (TEM) and high‐resolution TEM (HRTEM, Figure 2e–h). Ag500‐MoS_2_@Ni_3_S_2_/NF has a nanorod structure covered by nanosheets with a diameter of ≈200 nm and length of 2 µm. The HRTEM images in Figure 2g–i show that Ag‐MoS_2_@Ni_3_S_2_/NF has lattice spacings of 0.63 and 0.32 nm corresponding to the (002) and (004) planes of MoS_2_ as well as 0.21 and 0.28 nm corresponding to the (202) and (110) planes of Ni_3_S_2_.^[^ ^5^, ^35^ ^]^ The interface is formed mainly between the (002) plane of the MoS_2_ nanosheets and (202) plane of the Ni_3_S_2_ nanorods. The TEM and HRTEM images of the other implanted MoS_2_@Ni_3_S_2_/NF samples are depicted in Figures S9–S13, Supporting Information. The diameter of the nanowires is also 150–200 nm and the length is 1–2 µm. The (002) interplanar spacing of Ag‐doped MoS_2_ is slightly larger than that of pristine MoS_2_, whereas those of the plasma‐doped MoS_2_ is slightly smaller than that of the pristine MoS_2_. Since Ag has a larger radius, the lattice spacing of MoS_2_ increases slightly, while the lattice spacings of MoS_2_ doped with elements with smaller diameters decrease slightly in line with XRD. Besides, the interplanar spacing of Ni_3_S_2_ is basically unchanged, which is verified from the side that there is very little Ag plasma implantation into Ni_3_S_2_. The selected‐area electron diffraction pattern in Figure 2j also discloses a clear lattice composed of diffraction points indicative of excellent crystallinity in the MoS_2_ nanosheets. Figure 2k shows that Mo, Ag, S, and Ni are distributed evenly throughout the nanorods wrapped by nanosheets and the elemental maps of Zr500‐MoS_2_@Ni_3_S_2_/NF (Figure S9, Supporting Information) also disclose uniform distributions of Mo, S, Zr, and Ni. These results corroborate that the 2D structure of MoS_2_ is not destroyed by plasma implantation and MoS_2_@Ni_3_S_2_/NF is produced successfully.

**Figure 3:**
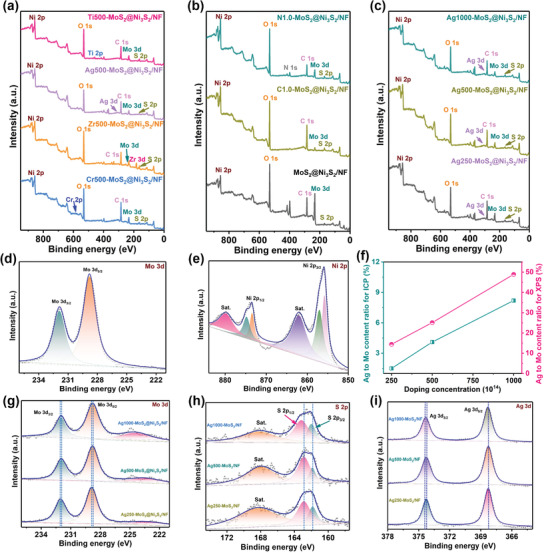
a,b) XPS survey spectra of MoS_2_@Ni_3_S_2_/NF samples; c) XPS survey spectra of MoS_2_@Ni_3_S_2_/NF with different dopant concentrations; XPS spectra of MoS_2_@Ni_3_S_2_/NF: d) Mo 3d and e) Ni 2p; f) Ag/Mo atomic ratios obtained by ICP and XPS; XPS spectra of Ag‐doped MoS_2_@Ni_3_S_2_/NF: g) Mo 3d, h) S 2p, and i) Ag 3d.

The XPS survey spectra in **Figure** **3**a–c show the presence of Mo, S, O, C, and Ni together with the dopants. The pure MoS_2_@Ni_3_S_2_/NF in Figure 3d displays two typical Mo 3d peaks at 228.7 and 231.9 eV corresponding to Mo 3d_5/2_ and Mo 3d_3/2_.^[^ ^36^ ^]^ The S 2p spectra in Figure S14, Supporting Information, show two peaks at 162.3 and 163.6 eV consistent with the spin split orbitals of S 2p_3/2_ and S 2p_1/2_, respectively^[^ ^37^ ^]^ and the Ni 2p spectrum of MoS_2_@Ni_3_S_2_ displays two pairs of peaks for Ni 2p_1/2_ (873.1 eV) and 2p_3/2_ (855 eV) associated with Ni^2+^ and Ni 2p_1/2_ (878.6 eV) and 2p_3/2_ (857.5 eV) stemming from Ni^3+^ (Figure 3e).^[^ ^38^ ^]^ Figure 3g presents the Mo 3d XPS spectra of Ag250‐MoS_2_@Ni_3_S_2_/NF, Ag500‐MoS_2_@Ni_3_S_2_/NF, and Ag1000‐MoS_2_@Ni_3_S_2_/NF. As the Ag concentration increases, the peaks of Mo 3d_3/2_ and Mo 3d_5/2_ shift to lower binding energies^[^ ^39^ ^]^ but the S 2p peaks shift toward higher binding energies (Figure 3h). The peaks of Ni 2p shifts to the direction of high binding energy with the increase of Ag doping, which demonstrates that the outer electron cloud density of Ni 2p decreases and Ni relatively loses electrons (Figure S15, Supporting Information). As shown in Figure 3i, the peaks of Ag 3d_5/2_ and Ag 3d_3/2_ remain at 368.3 and 374.3 eV with increasing Ag concentration^[^ ^40^ ^]^ and the ratios of Ag to Mo in Ag‐doped MoS_2_ are 14.4%, 25.1%, and 48.9% (Figure 3f) and larger than those determined by ICP, indicating that Ag is mainly concentrated on the surface of MoS_2_ and plays a role in the HER activity. The ratios of Ag to Mo obtained by ICP and XPS for increasing Ag concentrations are consistent as shown by a linear relationship. The XPS spectra of the other catalysts including Zr‐doped, Cr‐doped, Ti‐doped, C‐doped, and N‐doped MoS_2_@Ni_3_S_2_/NF are shown in Figures S16–S20, Supporting Information. The two peaks at 184.3 and 182.1 eV are Zr 3d_3/2_ and Zr 3d_5/2_ and Zr 2p peaks become more intense with increasing Zr concentrations.^[^ ^41^ ^]^ The binding energies of Ti 2p_1/2_ and Ti 2p_3/2_ remain at 464.3 and 458.4 eV and those of Cr 2p_1/2_ and Cr 2p_3/2_ are at 586.5 and 576.4 eV.^[^ ^42^, ^43^ ^]^ Similarly, with increasing metal dopant concentrations, the XPS peaks become clearer but the structure of MoS_2_ is not destroyed. However, the non‐metal dopants C and N show different results. The corresponding Mo 3d and S 2p peaks change when the C and N plasma implantation time is too long, implying that the MoS_2_ structure in the near surface is destroyed slightly.^[^ ^44^, ^45^ ^]^

### 2.2 Electrocatalytic Properties in HER

**Figure 4:**
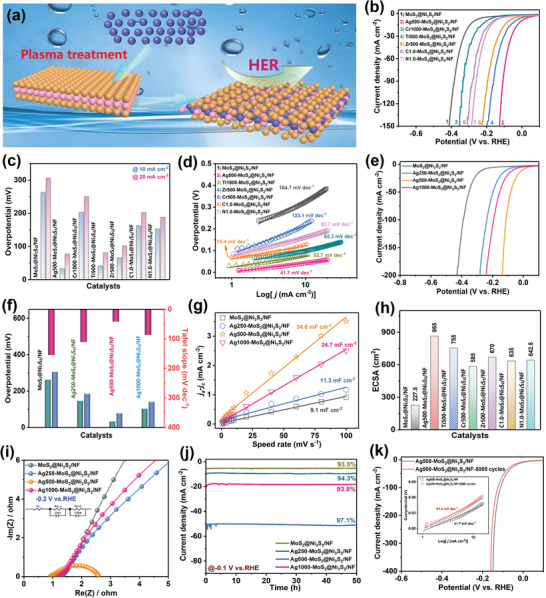
a) Schematic diagram showing the preparation of plasma doped MoS_2_ and HER characteristics on MoS_2_@Ni_3_S_2_/NF doped with different elements; b) LSV curves; c) overpotentials; d) Tafel plots; HER characteristics of Ag‐doped MoS_2_@Ni_3_S_2_/NF: e) LSV curves; f) overpotentials and Tafel plots; g) Capacitive current densities at different scanning rates; h) ECSA of different samples; i) Nyquist plots derived from EIS; j) Stability of Ag‐doped MoS_2_@Ni_3_S_2_/NF; k) Comparison of polarization curves.

The plasma‐implanted MoS_2_ shown in **Figure** **4**a swells slightly due to the large number of defects and more catalytic surface area which is likely to adsorb H_2_O or H atoms in HER. A standard three‐electrode system is adopted to evaluate the HER activity of the Ag‐doped MoS_2_@Ni_3_S_2_/NF electrode in 1 m KOH electrolyte. For comparison, the HER characteristics of the pure MoS_2_@Ni_3_S_2_/NF, Ag500‐MoS_2_@Ni_3_S_2_/NF, Cr1000‐MoS_2_@Ni_3_S_2_/NF, Zr500‐MoS_2_@Ni_3_S_2_/NF, Ti500‐MoS_2_@Ni_3_S_2_/NF, C1.0‐MoS_2_@Ni_3_S_2_/NF, and N1.0‐MoS_2_@Ni_3_S_2_/NF are also assessed under the same conditions. The iR‐corrected polarization curves of the samples are presented in Figure 4b. Ag500‐MoS_2_@Ni_3_S_2_/NF shows the best activity among the different samples. The overpotential (*η*) required for current densities (*j*) of 10 mA cm^−2^ (*η*_10_) and 20 mA cm^−2^ (*η*_20_) are merely 33 and 77 mV, respectively (Figure 4c). In comparison, *η*_10_ and *η*_20_ of MoS_2_@Ni_3_S_2_/NF (263 and 306 mV), Cr1000‐MoS_2_@Ni_3_S_2_/NF (173 and 220 mV), Zr500‐MoS_2_@Ni_3_S_2_/NF (66 and 112 mV), Ti500‐MoS_2_@Ni_3_S_2_/NF (43 and 86 mV), C1.0‐MoS_2_@Ni_3_S_2_/NF (163 and 202 mV), and N1.0‐MoS_2_@Ni_3_S_2_/NF (153 and 188 mV) are larger than those of Ag500‐MoS_2_@Ni_3_S_2_/N. The Tafel plots in Figure 4d confirm the improved HER activity. Compared to MoS_2_@Ni_3_S_2_/NF (164.7 mV dec^−1^), Cr1000‐MoS_2_@Ni_3_S_2_/NF (123.1 mV dec^−1^), Zr500‐MoS_2_@Ni_3_S_2_/NF (65.3 mV dec^−1^), Ti500‐MoS_2_@Ni_3_S_2_/NF (52.7 mV dec^−1^), C1.0‐MoS_2_@Ni_3_S_2_/NF (70.4 mV dec^−1^), and N1.0‐MoS_2_@Ni_3_S_2_/NF (85.7 mV dec^−1^), Ag500‐MoS_2_@Ni_3_S_2_/NF has smallest Tafel slope (41.7 mV dec^−1^) suggesting better S activation on the MoS_2_ basal surface and the Volmer–Heyrovsky mechanism.^[^ ^46^ ^]^ The Volmer–Heyrovsky process is shown in Figure S21, Supporting Information.

The Ag concentration is important to the catalytic effects of the MoS_2_ nanosheets and Ni_3_S_2_ nanorods. Therefore, three samples implanted with different Ag concentrations, namely Ag250‐MoS_2_@Ni_3_S_2_/NF, Ag500‐MoS_2_@Ni_3_S_2_/NF, and Ag1000‐MoS_2_@Ni_3_S_2_/NF, are studied and the corresponding polarization curves are shown in Figure 4e. As the Ag concentration increases, the catalytic activity goes up initially and then decreases and Ag500‐MoS_2_@Ni_3_S_2_/NF exhibits the best HER activity. Moreover, Ag500‐MoS_2_@Ni_3_S_2_/NF shows a Tafel slope of 41.7 mV dec^−1^ which is less than those of MoS_2_@Ni_3_S_2_/NF (164.7 mV dec^−1^), Ag250‐MoS_2_@Ni_3_S_2_/NF (110.5 mV dec^−1^), and Ag1000‐MoS_2_@Ni_3_S_2_/NF (86.4 mV dec^−1^) (Figure 4f and Figure S22, Supporting Information). The small Tafel slope of Ag500‐MoS_2_@Ni_3_S_2_/NF suggests a large number of active sites. Ag500‐MoS_2_@Ni_3_S_2_/NF requires an overpotential of only 33 mV for a current density of 10 mA cm^−2^, which is 230, 113, and 71 mV less than those of the bare MoS_2_@Ni_3_S_2_/NF, Ag250‐MoS_2_@Ni_3_S_2_/NF and Ag1000‐MoS_2_@Ni_3_S_2_/NF (Figure 4f).

Electrochemical impedance spectroscopy (EIS) is an important technique to evaluate the catalytic activity of electrocatalysts. As shown in Figure 4i and Table S2, Supporting Information, Ag500‐MoS_2_@Ni_3_S_2_/NF has the smallest charge transfer resistance *R*_ct dl_ (Ω) and *R*_s_ (Ω) implying higher conductivity and more channels for faster ion transfer. For comparison, the EIS data of the other plasma‐doped MoS_2_@Ni_3_S_2_/NF samples acquired at −0.1 V versus RHE are shown in Figure S23, Supporting Information. After plasma implantation, MoS_2_ shows a relatively small impedance and better conductivity, which facilitate transport of electrons and ions in HER. To further investigate the working range and effectiveness of plasma implantation, different amounts of Zr, Ti, Cr, N, and C are implanted into MoS_2_@Ni_3_S_2_/NF. The electrocatalytic activity of Zr‐doped MoS_2_@Ni_3_S_2_/NF, Ti‐doped MoS_2_@Ni_3_S_2_/NF, N‐doped MoS_2_@Ni_3_S_2_/NF, Cr‐doped MoS_2_@Ni_3_S_2_/NF, and C‐doped MoS_2_@Ni_3_S_2_/NF in HER is assessed under the same conditions. The polarization curves after iR correction in Figures S24–S28, Supporting Information, show that plasma implantation improves the catalytic activity compared to the pristine MoS_2_@Ni_3_S_2_/NF. The activity rises in the beginning and then drops as the dopant concentration increases. The Tafel slopes of plasma‐doped Ti500‐MoS_2_@Ni_3_S_2_/NF (Figure S24b, Supporting Information), Zr500‐MoS_2_@Ni_3_S_2_/NF (Figure S25b, Supporting Information), N1.0‐MoS_2_@Ni_3_S_2_/NF (Figure S26b, Supporting Information), Cr500‐MoS_2_@Ni_3_S_2_/NF (Figure S27b, Supporting Information), and C1.0‐MoS_2_@Ni_3_S_2_/NF (Figure S28b, Supporting Information) are 52.7, 65.3, 116.5, 80.4, and 71.4 mV dec^−1^, suggesting better HER characteristics compared with other samples. However, the Tafel slopes of Ti250‐MoS_2_@Ni_3_S_2_/NF, Zr250‐MoS_2_@Ni_3_S_2_/NF, Cr250‐MoS_2_@Ni_3_S_2_/NF, C2.0‐MoS_2_@Ni_3_S_2_/NF, and N4.0‐MoS_2_@Ni_3_S_2_/NF are 121.2, 135.3, 151.4, 147.2, and 111.7 mV dec^−1^ and not as good as that of the original MoS_2_@Ni_3_S_2_/NF (164.7 mV dec^−1^). Hence, the proper amount of dopants is crucial to the HER performance. Moreover, the *η*_10_ values of Ti500‐MoS_2_@Ni_3_S_2_/NF, Zr500‐MoS_2_@Ni_3_S_2_/NF, Cr500‐MoS_2_@Ni_3_S_2_/NF, C1.0‐MoS_2_@Ni_3_S_2_/NF, and N1.0‐MoS_2_@Ni_3_S_2_/NF at 10 mA cm^−2^ are 43, 66, 174, 125, 100 mV, which are 76, 112, 96, 82, and 86 mV lower than those of Ti250‐MoS_2_@Ni_3_S_2_/NF (Figure S24c, Supporting Information), Zr250‐MoS_2_@Ni_3_S_2_/NF (Figure S25c, Supporting Information), N4.0‐MoS_2_@Ni_3_S_2_/NF (Figure S26c, Supporting Information), Cr250‐MoS_2_@Ni_3_S_2_/NF (Figure S27c, Supporting Information), and C0.5‐MoS_2_@Ni_3_S_2_/NF (Figure S28c, Supporting Information), respectively. In addition, comparison of *η*_20_ at the larger current density (20 mA cm^−2^) shows the same trend as that at 10 mA cm^−2^. The overpotential of the original MoS_2_@Ni_3_S_2_/NF is higher than that of the plasma‐doped catalysts, and the overpotential of the Ag and Ti plasma‐doped catalyst is smaller than those of the other samples, indicating that plasma doping improves the catalytic activity of MoS_2_.

The electrochemical surface area (ECSA) is derived from the CV curves (Figures S29 and S30, Supporting Information). The electric double layer capacitance (EDLC) of Ag500‐MoS_2_@Ni_3_S_2_/NF is 34.6 mF cm^−2^ (Figure 4g) which is significantly bigger than those of MoS_2_@Ni_3_S_2_/NF (9.1 mF cm^−2^), Ag250‐MoS_2_@Ni_3_S_2_/NF (11.3 mF cm^−2^), and Ag1000‐MoS_2_@Ni_3_S_2_/NF (24.7 mF cm^−2^). The large EDLC demonstrates that Ag500‐MoS_2_@Ni_3_S_2_/NF has a larger electrochemically active area for HER and more active sites. The EDLCs of the other electrodes with different dopant concentrations are shown in Figure S31, Supporting Information. The EDLCs of Zr500‐MoS_2_@Ni_3_S_2_/NF, Cr500‐MoS_2_@Ni_3_S_2_/NF, Ti500‐MoS_2_@Ni_3_S_2_/NF, C1.0‐MoS_2_@Ni_3_S_2_/NF, and N1.0‐MoS_2_@Ni_3_S_2_/NF are 26.8, 23.4, 30.2, 25.4, and 25.7 mF cm^−2^, respectively, with the following order: Ag > Ti > Zr > N > C > Cr. The ECSAs of the Ag‐doped samples is larger than those of other dopants (Figure 4h). Again, the catalytic activity increases and then decreases with dopant concentrations consistent with the Tafel slopes and overpotentials.

The electrochemical stability of the catalysts is evaluated by chronoamperometry and cyclic voltammetry (CV). As shown in Figure 4j, compared to Ag1000‐MoS_2_@Ni_3_S_2_/NF and Ag250‐MoS_2_@Ni_3_S_2_/NF with too much or too little Ag dopants, the Ag500‐MoS_2_@Ni_3_S_2_/NF exhibits not only excellent initial HER activity, but also good electrocatalytic durability. After 50 h, the catalyst retains 97.1% of the activity with basically no decrease in the current density. Moreover, Ag500‐MoS_2_@Ni_3_S_2_/NF shows negligible loss in the polarization current density after 5000 cycles and compared with the Tafel slope and EIS data of the initial electrode, the results are similar (Figure 4k and Figure S32, Supporting Information) confirming that Ag500‐MoS_2_@Ni_3_S_2_/NF has excellent catalytic stability. Chronoamperometry is also performed on Zr‐doped MoS_2_@Ni_3_S_2_/NF, Ti‐doped MoS_2_@Ni_3_S_2_/NF, Cr‐doped MoS_2_@Ni_3_S_2_/NF, C‐doped MoS_2_@Ni_3_S_2_/NF, and N‐doped MoS_2_@Ni_3_S_2_/NF for HER (Figure S33, Supporting Information). A fluence of 5 × 10^16^ ions cm^−2^ shows the best stability and the current density retention is basically close to 90% after cycling for 30 h, for example, Zr500‐MoS_2_@Ni_3_S_2_/NF (97.1%), Ti500‐MoS_2_@Ni_3_S_2_/NF (88.9%), and Cr500‐MoS_2_@Ni_3_S_2_/NF (89.3%). The electrodes implanted with a fluence of 2.5 × 10^16^ ions cm^−2^ has the worst catalytic stability and the current density retention is only 80%, for instance, Zr250‐MoS_2_@Ni_3_S_2_/NF (83.3%), Ti250‐MoS_2_@Ni_3_S_2_/NF (78.8%), and Cr250‐MoS_2_@Ni_3_S_2_/NF (84.7%). For the non‐metallic plasma‐doped samples, the samples treated for one hour show better cycle stability compared to those treated for 0.5, 2, and 4 h. After applying −0.15 V for 30 h, the current densities of C1.0‐MoS_2_@Ni_3_S_2_/NF and N1.0‐MoS_2_@Ni_3_S_2_/NF are maintained at more than 90% of the initial value, but that of N4.0‐MoS_2_@Ni_3_S_2_/NF drops to 78%, indicating that the long‐term stability of the carbon or nitrogen‐doped samples increases and then decreases with implantation time as well.

### 2.3 Theoretical Assessment

**Figure 5:**
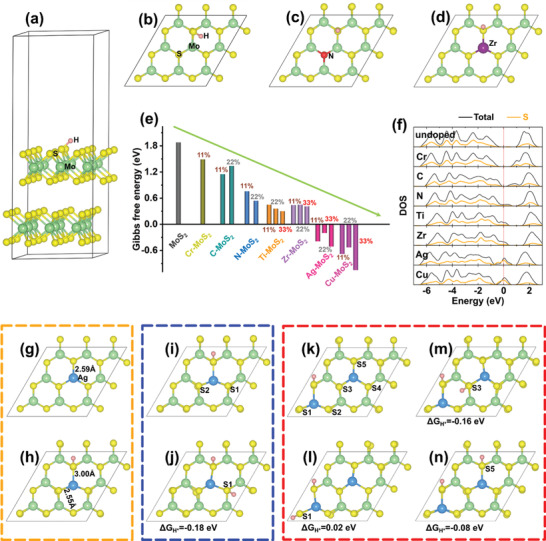
a) Optimized structure of H* adsorption on bare MoS_2_(002); Top views of H* adsorption on b) bare MoS_2_; c) N—MoS_2_, and d) Zr—MoS_2_; e) Calculated Δ*G*_H*_ of MoS_2_ doped with different elements; f) DOS of S sites on MoS_2_ doped with different elements; g,h) Changes of Ag—S bond lengths before and after H* adsorption; i,j) Top‐view schematics of different H* coverages on 11 mol% Ag‐MoS_2_; k–n) Top‐view schematic of different H* coverages on 22 mol% Ag‐MoS_2_.

The HER sites are concentrated on the surface of the catalyst and in this respect, the plasma‐modified MoS_2_ forms the outermost layer on the catalyst. The change of the Gibbs free energy (Δ*G*_H*_) between adsorption and desorption of H* on the electrode surface is a key parameter to predict the HER activity and the closer Δ*G*_H*_ is to zero, the higher is the HER efficiency.^[^ ^42^, ^47^, ^48^ ^]^ Therefore, Δ*G*_H*_ for H* adsorbed on the plasma‐treated MoS_2_ surface is first evaluated because H* tends to adsorb onto the sulfur (S) sites.^[^ ^49^, ^50^ ^]^ The supercell with the 3 × 3 surface unit cells and top‐view H* adsorption above the S sites is shown in **Figure** **5**a,b. In the first‐principles calculation, H* adsorbed on the S sites of the Cr‐, Ti‐, Zr‐, Ag‐, Cu‐, C‐, and N‐doped MoS_2_ surfaces is derived separately for three dopant concentrations of 11, 22, and 33 mol% according to the XPS data (Figure 3f). The top‐views of 11 mol% Zr‐ and N‐doped MoS_2_ are depicted in Figure 5c,d, respectively, and the calculated Δ*G*_H*_ values of the series of MoS_2_ samples implanted with different elements are shown in Figure 5e. In order to exclude the surface of Ag‐Ni_3_S_2_ as potential active sites, we also calculated the hydrogen adsorption energy on its (110) and (202) surfaces, respectively, and compared them with the hydrogen adsorption energy calculated on the Ag‐MoS_2_ surface (Figure S35, Supporting Information), which demonstrated that the catalytic activity on the surface of Ag‐MoS_2_ is better, and it is more reasonable as the active site. Δ*G*_H*_ of 22% Ag‐doped MoS_2_ is closer to zero than the other samples, especially pure MoS_2_ (1.88 eV), indicating that plasma doping can indeed raise the HER activity of the S sites on the basal surface. There is a relationship between Δ*G*_H*_ of MoS_2_ doped with transition metals (Ag, Cu, Ti, Zr, Cr) and the number of valence electrons of the dopants. The smaller the number of valence electrons, the lower is Δ*G*_H*_ at this site, implying that metal dopants can more effectively activate S at the replacement sites. The effects of impurity atoms on valence electrons are also revealed by the density of states (DOSs) in Figure 5f. The valence band of pure MoS_2_ is full and the valence orbital of the S atom is saturated. Therefore, it is difficult for H* to adsorb to the S site thereby giving rise to a larger Δ*G*_H*_.^[^ ^51^ ^]^

When dopants with less valence electrons than Mo are incorporated, empty states emerge from the valence band and the smaller the number of valence electrons, the larger is the number of empty states. Accordingly, as shown by the DOS of S atoms in the plasma‐treated MoS_2_ in Figure 5f, introduction of metal dopants with less electrons makes the valence orbitals of S atoms unsaturated and the empty states in the valence orbitals also increase, so that it is relatively easy for H* to adsorb to the active sites of S and Δ*G*_H*_ is closer to zero. In contrast, Cr has the same valence electrons as Mo and so Δ*G*_H*_ of Cr‐doped MoS_2_ is similar to that of pure MoS_2_. Regarding non‐metallic dopants, N doping produces empty states in the valence orbitals of S leading to a lower Δ*G*_H*_ than undoped MoS_2_, whereas C doping does not produce empty states in the valence band and consequently a large Δ*G*_H*_.

Structural strain is also an important factor for Δ*G*_H*_ because the valence state of the substituted metal atom is lower and the bond with the adjacent S atom is weaker than that of the Mo—S bond.^[^ ^52^, ^53^ ^]^ As shown in Figure 5g, the Ag—S bond length (2.59 Å) is larger than the Mo—S bond length (2.42 Å) before H adsorption on S, leading to stretch strain in the bond between the impurity atom and S. As shown in Figure 5h, the bond length between the Ag atom directly connected to the H*‐adsorbed S atom increases from 2.59 to 3.00 Å, while the other Ag—S bond length decreases from 2.59 to 2.55 Å, implying that H adsorption releases this strain and therefore lowers Δ*G*_H*_. Δ*G*_H*_ in Figure 5e corresponds to adsorption of one H atom on one S atom which directly bonds with impurity metal atoms as shown in Figure 5d,h. Δ*G*_H*_ at the S site that does not bond with metal atoms is higher than zero (Figure S36, Supporting Information), meaning that the S site that directly bonds with the impurity atom is the active site for hydrogen evolution, while the S site far away from the impurity atom is basically inactive. Therefore, the S atoms that do not bond with the metal atoms will not be discussed in the following section.

It has been shown that the dopant concentration in the catalyst plays an important role in adjusting Δ*G*_H*_.^[^ ^54^ ^]^ Figure 5e displays the Δ*G*_H*_ values of C, N, Ti, Zr, Ag, and Cu plasma‐doped MoS_2_ for concentrations of 11, 22, and 33 mol%. As the dopant concentration increases from 11 to 33 mol%, Δ*G*_H*_ approaches zero initially and then increases, especially Ag, Cu, Ti doped MoS_2_, proving that more adjacent S active sites are activated as the dopant concentration goes up consistent with the experimental results. However, if the dopant concentration increases to 33%, Δ*G*_H*_ of the S active site moves away from the zero point. Therefore, the optimal dopant concentration for activation of adjacent S sites is 22% (Figure S37, Supporting Information). With regard to Ag‐doped MoS_2_ with concentration of 11% and 22%, the Δ*G*_H*_ values of one H* adsorbed on the active S site is −0.39 and −0.2 eV (monolayer H coverage is 11%), which are below zero suggesting that H* can easily adsorb on active S but desorption is relatively difficult. There is thus a certain amount of H atoms adsorbed on the surface of the active site during HER and so calculation of the Gibbs free energy for multiple hydrogen adsorption on the active S site is important. Therefore, Δ*G*_H*_ for additional H atom adsorption for 11 mol% H coverage and Δ*G*_H*_ for second H* adsorption after one H* has already adsorbed to the 3 × 3 surface unit cell are derived. Here, Δ*G*_H*_ is defined as follows: Δ*G*_H*_ = *E*_surf+2H*_ − (*E*_surf+H*_ + 0.5*E*_H2_) + 0.24 eV, where *E*_surf+H*_ is the total energy of the super cell with one adsorbed H* and *E*_surf+2H*_ is the total energy of the supercell with two adsorbed H*.^[^ ^55^ ^]^ The active S site on the surface of 11 mol% Ag‐doped MoS_2_ is occupied by one H* (Figure 5i) and there are still two equivalent activated S atoms that can adsorb the second H*. Figure 5j exhibits the surface structure for second H* adsorption and Δ*G*_H*_ for second H* adsorption is only −0.18 eV. With increasing H coverage from 11 to 22%, Δ*G*_H*_ rises from −0.39 to −0.18 eV because adsorption of the first H atom decrease the empty states of the valence orbitals of the other S atom. Hence, adsorption of the second H atom becomes weak. As for the 22 mol% Ag doped MoS_2_ surface with 11% H coverage in Figure 5k, there are five analogous activated S atoms to adsorb the second H atom. Figure 5l–n exhibit the three typical configurations of two H* adsorbed on the 22% Ag doped MoS_2_ surface together with the corresponding Δ*G*_H*_ of the second H*. Δ*G*_H*_ for adsorption of the second H* atom increases compared to that for the first H*. Remarkably, Δ*G*_H*_ for the two S active sites connected to the same Ag in Figure 5l is only 0.02 eV and closer to zero, indicating that there are more high active sites on the 22% Ag‐doped MoS_2_ surface. The main reason is that the two H* atoms adsorbed on the two S atoms are connected to the same Ag atom (Figure 5l) and two H* atoms adsorb to two S atoms that connect directly with the Ag atoms at two different positions (Figure 5m,n). As aforementioned, direct adsorption of H* atoms on S atoms bonded to the same Ag atom can release more strain energy in the Ag—S bond. The first H* adsorption releases the main stain energy in the Ag—S bond, whereas the second H* adsorption releases less strain energy and shows a larger Δ*G*_H*_. The theoretical results are supported by the experimental data acquired from Ag‐doped MoS_2_. All in all, the proper dopant and fluence create more S orbital empty states to promote the HER activity of MoS_2_.

### 2.4 Overall Water Splitting

**Figure 6:**
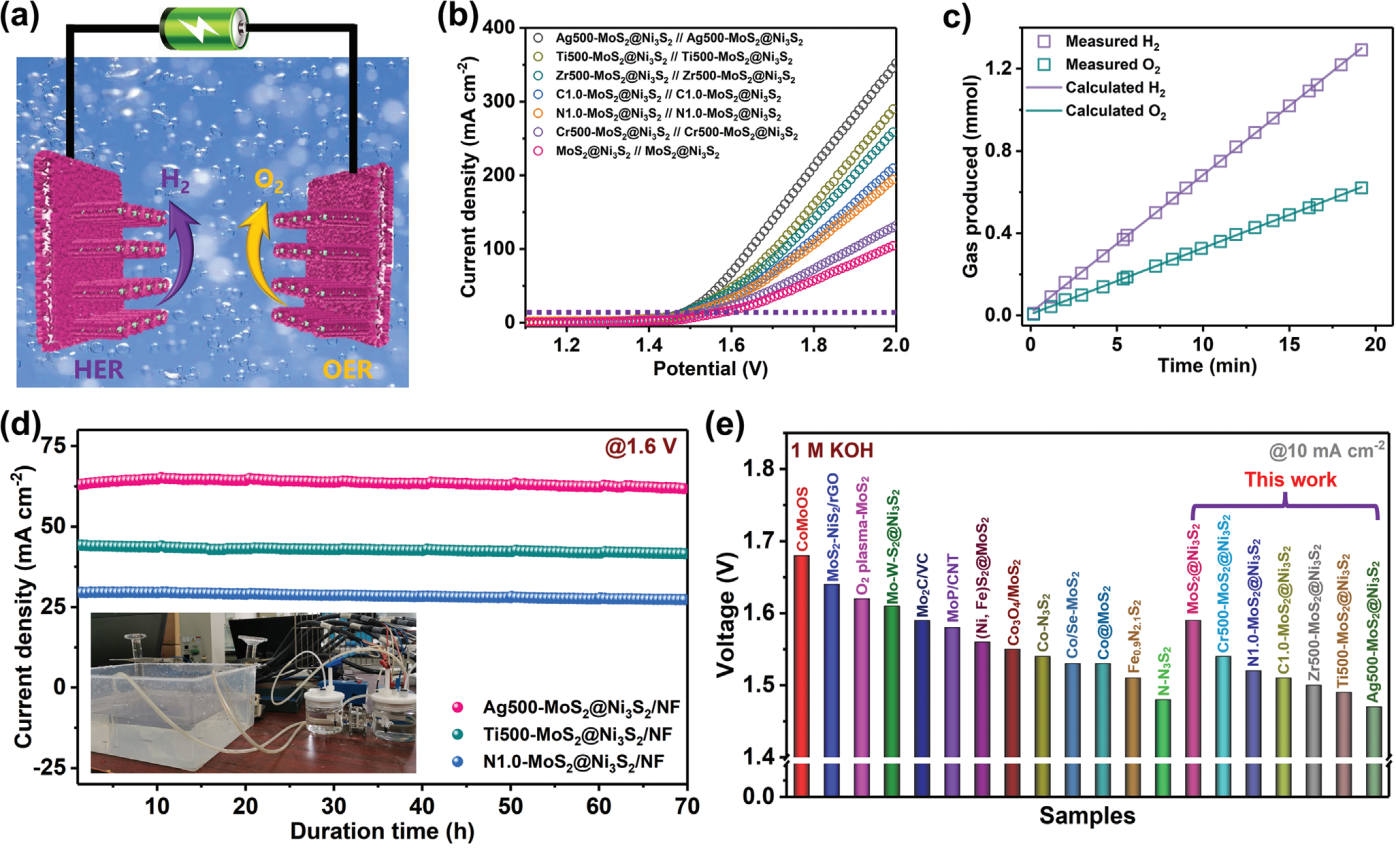
a) Schematic diagram of the water electrolysis apparatus with two electrodes; b) two‐electrode polarization curves obtained at a scanning rate 5 mV s^−1^; c) Comparison of the experimental and theoretical amount of H_2_ and O_2_ produced in overall water splitting; d) Chronoamperometry of overall water splitting at 1.6 V; e) Comparison of the cell voltages to achieve 10 mA cm^−2^ for recently reported electrocatalysts.

As the other half‐reaction in water splitting, the OER catalytic activity of doped MoS_2_@Ni_3_S_2_/NF is also assessed in the same alkaline electrolyte. Figure S38a, Supporting Information, shows the polarization curves of Ag500‐MoS_2_@Ni_3_S_2_/NF, Zr500‐MoS_2_@Ni_3_S_2_/NF, N1.0‐MoS_2_@Ni_3_S_2_/NF, Ti500‐MoS_2_@Ni_3_S_2_/NF, C1.0‐MoS_2_@Ni_3_S_2_/NF, bare MoS_2_@Ni_3_S_2_/NF and corresponding Tafel slopes and overpotentials are shown in Figure S38b, Supporting Information. Compared with other electrodes, the Ag500‐MoS_2_@Ni_3_S_2_/NF catalytic electrode still has a relatively small OER Tafel slope and overpotential, showing outstanding catalytic activity for OER. Owing to the excellent HER and OER catalytic properties, MoS_2_@Ni_3_S_2_/NF is adopted as both the anode and cathode in a two‐electrode system with an alkaline electrolyte (Figure S39, Supporting Information) as illustrated in **Figure** **6**a. As shown in the polarization curve of the two electrodes in Figure 6b, Ag500‐MoS_2_@Ni_3_S_2_/NF||Ag500‐MoS_2_@Ni_3_S_2_/NF only needs 1.47 V to drive a current density of 10 mA cm^−2^, which is far less than that of Ti500‐MoS_2_@Ni_3_S_2_/NF||Ti500‐MoS_2_@Ni_3_S_2_/NF (1.49 V), Zr500‐MoS_2_@Ni_3_S_2_/NF||Zr500‐MoS_2_@Ni_3_S_2_/NF (1.5 V), C1.0‐MoS_2_@Ni_3_S_2_/NF||C1.0‐MoS_2_@Ni_3_S_2_/NF (1.51 V), N1.0‐MoS_2_@Ni_3_S_2_/NF||N1.0‐MoS_2_@Ni_3_S_2_/NF (1.52 V), Cr500‐MoS_2_@Ni_3_S_2_/NF||Cr500‐MoS_2_@Ni_3_S_2_/NF (1.55 V), and bare MoS_2_@Ni_3_S_2_/NF||MoS_2_@Ni_3_S_2_/NF (1.6 V). In particular, the driving voltage of the original MoS_2_@Ni_3_S_2_/NF electrode is 1.6 V, which is much larger than those of other electrodes modified by the plasma.

Faraday efficiency is another important parameter to evaluate the efficiency of the electrocatalytic reaction and the gas volume is compared to the theoretical value. Figure 6c shows that the experimental and theoretical values for HER and OER of the Ag500‐MoS_2_@Ni_3_S_2_@NF electrode are basically the same approaching 100% and a fairly stable hydrogen/oxygen yield is achieved. The long‐term stability of the two electrodes is another important parameter in practice. The overall water splitting apparatus with two electrodes is shown in the inset in Figure 6d and the long‐term durability of Ag500‐MoS_2_@Ni_3_S_2_/NF, Ti500‐MoS_2_@Ni_3_S_2_/NF, and N1.0‐MoS_2_@Ni_3_S_2_/NF is assessed by continuous operation at 1.6 V in 1 m KOH. The three plasma‐doped electrodes maintain the stability for 70 h without appreciable attenuation (Figure 6d) confirming the important effects of the plasmas. Figure S40, Supporting Information, shows that after the HER stability test, the morphology of the entire electrode is intact and the TEM image shows that the crystal lattice is not damaged. At the same time, Mo, Ni, Ag, and S are still present as shown by EDS. As shown in Figure S41, Supporting Information, the change in the valence state of Ag in the sample is basically negligible, and it mainly plays a role in activating the S site. However, the peak positions of S 2p and Ni 2p have changed a lot, which is attributed to the stability test in an alkaline environment, and part of the S and Ni sites are oxidized. It should be emphasized that the overall water splitting performance of Ag500‐MoS_2_@Ni_3_S_2_/NF exceeds that of recently reported Mo‐based electrocatalysts (Figure 6f). Our results and analysis reveal that plasma engineering activates MoS_2_@Ni_3_S_2_ leading to enhanced and durable HER activity.

## 3 Conclusion

MoS_2_@Ni_3_S_2_ is subjected to plasma implantation of metallic and non‐metallic elements with different fluences to modify the surface chemistry and enhance the hydrogen evolution activity. Ag500‐MoS_2_@Ni_3_S_2_/NF exhibits the best catalytic activity and durability for HER in an alkaline environment. First‐principles calculation reveals that the Gibbs free energy for H atom adsorption on the Ti‐, Zr‐, Ag‐, and Cu‐doped MoS_2_ surfaces are lower than that on the undoped MoS_2_ surface. The smaller Gibbs free energy stems from empty states in the valence band of MoS_2_ introduced by these dopants, leading to stronger bonds between S and adsorbed H atoms. Δ*G*_H*_ of 22 mol% Ag‐doped MoS_2_ is only 0.02 eV and close to zero resulting in high HER efficiency. The two‐electrode water splitting system with the Ag500‐MoS_2_@Ni_3_S_2_/NF anode and cathode needs a battery voltage of 1.47 V to generate a current density of 10 mA cm^−2^. Our results reveal the benefits of plasma engineering and sheds light into the associated mechanisms, which are beneficial to the design and fabrication of transition metal electrocatalysts with high efficiency and robust stability.

## 4 Experimental Section

### Preparation of MoS_2_@Ni_3_S_2_/NF

0.3 mmol ammonium molybdate and 12 mmol thiourea were added into 50 mL of DI water separately and stirred for 15 min to form a clear solution. The colorless solution and 2 cm × 3 cm clean NF were separately transferred to a hydrothermal reactor maintained at 200 °C for 12 h. After the hydrothermal reaction, the samples were taken out and rinsed with absolute ethanol and DI water for 5 min to remove impurities. Finally, the samples were put in a vacuum oven at 60 °C overnight to dry and prevent surface oxidation.

### Plasma Injection MoS_2_@Ni_3_S_2_/NF

A high‐energy metal ion plasma system and a multifunctional gas plasma system were used (Supporting Information). The Ag plasma process used to treat MoS_2_@Ni_3_S_2_/NF is described here as an example. The Ag target was polished mechanically. The MoS_2_@Ni_3_S_2_/NF sample and Ag target were transferred to the PI‐80A high‐energy metal plasma system with a base pressure of 2 × 10^−3^ Pa and Ag plasma ion implantation was performed at an accelerating voltage of 25 kV with a fluence of 5 × 10^16^ ions cm^−2^ on MoS_2_@Ni_3_S_2_/NF (Ag500‐MoS_2_@Ni_3_S_2_/NF). In addition, 2.5 × 10^16^ and 1 × 10^17^ ions cm^−2^ Ag ions were implanted into MoS_2_@Ni_3_S_2_/NF for comparison (Ag250‐MoS_2_@Ni_3_S_2_/NF and Ag1000‐MoS_2_@Ni_3_S_2_/NF). The other plasma targets employed to implant MoS_2_@Ni_3_S_2_ included Zr, Ti, Cr, C, and N plasma. The plasma implantation process was similar to that for Ag and three fluences of each element were implanted for comparison. The schematic diagram of the metal plasma and non‐metal plasma doping processes is shown in Figure S1, Supporting Information. Compared with the initial NF, the mass loading of the entire Ag500‐MoS_2_@Ni_3_S_2_/NF catalytic electrode is 7.13 mg cm^−2^.

### Materials Characterization

The surface morphology, size, and elemental composition were characterized by field‐emission scanning electron microscopy (Zeiss Gemini 450, Germany) equipped with energy‐dispersive X‐Ray spectrometry (EDS). The phase composition was analyzed by XRD (Rigaku, RINT2100, Japan) with Cu K_*α*_ radiation (*λ* = 1.5406 Å) and TEM and HRTEM were conducted to examine the fine structure and lattice spacing on the JEOL JEM‐2010. The elemental maps were acquired by EDS on the SEM and TEM and the elemental concentrations were also determined by ICP‐AES (Agilent, USA). The BET specific surface area was determined on the automatic specific surface and pore size distribution analyzer (Quantachrome Instruments, USA) and the surface chemistry was analyzed by X‐ray photoelectron spectroscopy (150W, ESCALAB 250) with Al K_*α*_ radiation.

### Electrochemical Measurements

The electrocatalytic tests were performed using the three‐electrode configuration on the VMP3 (Bio‐Logic) in 1 m KOH at room temperature. The sample, saturated calomel electrode, and graphite rod were the working, reference, and counter electrodes, respectively. Linear sweep voltammetry (LSV) was carried out at a scanning rate of 5 mV s^−1^. The potentials in the LSV polarization curves were iR‐corrected with respect to the ohmic resistance of the solution and the current densities were based on the geometrical area. The ECSA of the electrode was determined by CV in the non‐faradaic potential region at different sweeping rates to derive the double layer capacitance (*C*_dl_) of the catalysts. EIS was conducted at the open circuit and certain potentials in the frequency range from 1 MHz to 0.1 Hz. The overall water splitting experiment was performed in a two‐electrode system with two symmetrical electrodes as the anode and cathode.

### First‐Principles Calculation

In the electrochemical HER, the efficiency depends mainly on the change in the Gibbs free energy (Δ*G*_H*_) between adsorption and desorption of H atoms on the electrode surface. The closer is Δ*G*_H*_ approaching zero, the higher is the HER efficiency.^[^ ^55^, ^56^ ^]^ To investigate how Δ*G*_H*_ on MoS_2_ surface varies with the different dopants (C, N, Cr, Ti, Zr, Ag, and Cu), Δ*G*_H*_ and H coverage was derived by first‐principles calculation (Supporting Information). The MoS_2_ surface was modeled with a supercell with 3 × 3 surface unit cell (Figure S34, Supporting Information). If only the last step of the reaction was considered and the desorption state of H_2_ was regarded as the zero point, the Tafel process and Heyrovsky process show different reaction details, but the Gibbs free energy changes during the process are the same. Δ*G*_H*_ is defined as Δ*G*_H*_ = *E*_surf+H*_ − (*E*_surf_ + 0.5*E*_H2_) + 0.24 eV,^[^ ^57^, ^58^ ^]^ where *E*_surf_ is the total energy of the supercell without any adsorbed H atom, *E*_surf+H*_ is the total energy of the supercell with an adsorbed H atom, *E*_H2_ is the binding energy of a H_2_ molecule, and 0.24 eV is the change of zero energy and heat of entropy change between adsorption and desorption of a H atom on the electrode surface.^[^ ^59^ ^]^

## Conflict of Interest

The authors declare no conflict of interest.

## Supporting information

Supporting Information

## Data Availability

Research data are not shared.
