# Supplementary material for: Plasma Engineering of Basal Sulfur Sites on MoS2@Ni3S2 Nanorods for the Alkaline Hydrogen Evolution Reaction
Source: Adv Sci (Weinh). 2021 Dec 22;9(6):2104774. doi: 10.1002/advs.202104774 (PMC8867165; doi:10.1002/advs.202104774)
Supplement: Supplementary file 1 — Supporting Information [file ADVS-9-2104774-s001.pdf]

WILEY-VCH

ADVANCED SCIENCE
Open Access

Supporting Information

for *Adv. Sci.*, DOI: 10.1002/advs.202104774

Plasma Doping Engineering Enhancing Basal S sites in MoS_2@Ni_3S_2 Heterogeneous Nanorods for Alkaline Hydrogen Evolution Reaction

Xin Tong★, Yun Li★, Qingdong Ruan, Ning Pang, Yang Zhou, Dajun Wu, Dayuan Xiong, Shaohui Xu, Lianwei Wang *, Paul K. Chu**

Supporting Information

Plasma Doping Engineering Enhancing Basal S sites in MoS_2@Ni_3S_2 Heterogeneous Nanorods for Alkaline Hydrogen Evolution Reaction

Xin Tong★, Yun Li★, Qingdong Ruan, Ning Pang, Yang Zhou, Dajun Wu*, Dayuan Xiong, Shaohui Xu, Lianwei Wang *, Paul K. Chu*

1.1 Introduction to Plasma Injection

Plasma injection is to first ionize the gas or metal to generate plasma, and then accelerate the target ions and act on the surface of the material for modification. Generally, it is low-temperature plasma, and the pressure is controlled at 10^{-3} ~ 10^{-1}. The plasma generation methods include DC glow discharge, radio frequency discharge, microwave discharge, and arc discharge. The amount of ion implantation is positively related to the current. The larger the current, the more ions reach the sample, and the energy of the ions is positively related to the applied voltage. The higher the applied voltage, the higher the energy of the ions. For this plasma injection process, we use low temperature plasma, so the temperature is not very high, just make sure the ion can be implanted in the sample, and make sure the ion energy is not very high and do not destroy the structure of the sample.

1.2 The difference between gas and high energy metal ion implantation system

The gas ion implantation system and the high-energy metal ion implantation system work differently. The high-energy ion implantation system accelerates the metal charged ions linearly, and the accelerated ions reach the sample surface. For the high-energy ion implantation system, firstly, the Ag target is ionized by arc discharge to obtain Ag plasma. Secondly, Ag plasma is accelerated by a 25 kV acceleration grid to obtain higher energy and accelerate to reach the sample surface on the grounded sample stage. Because of its relatively large energy, a certain depth (tens to hundreds of nanometers) can be implanted on the sample, depending on the set high pressure value and the characteristics of the sample. For the gas ion implantation system, a negative high voltage is connected to the sample, and the positively charged ions in the vacuum chamber are attracted to the sample.

1.3 Calculation method

The first-principles calculations are carried out by using VASP code, in which the generalized-gradient-approximation (GGA)[S1] exchange-correlation functional[S2] and the projector augmented wave method[S3] are used. The cutoff kinetic energy for the plane wave basis set is 450 eV. To correctly evaluate the strong on-site Coulomb repulsion among the electrons in transition metal atoms 3d or 4d orbitals, GGA+U method[S4] is applied in the calculations, in which (U-J) = 2.4 eV for Mo and Zr 4d orbitals, and (U-J) = 3.7 eV for Ti 3d orbitals. The MoS_2 surface is modelled in a supercell shown in **Figure S33**, which consists of two (3 × 3)-unit-cell layers and a vacuum layer of ~13Å. A Gamma-type 5 × 5 × 1 k-point mesh is applied to sample the Brillouin zone. All atoms are fully relaxed until the forces on the atoms are less than 0.02 eV/Å. We simulated the Gibbs free energy changes in MoS_2 at three doping concentrations of 11 mol%, 22 mol%, and 33 mol%.

Figures S34 displays the surface atomic structures of 11 mol% Ag-doped MoS_2 and N-doped MoS_2. **Figure S35** displays ΔG_{H*} of H adsorption on the S atoms at different sites with respect to the impurity atoms. For all the metal elements doping the ΔG_{H*} on the 1st nearest neighbor S atoms of the impurity atoms, the S atoms which bond with the impurity atoms, is remarkably lower than the 2nd and 3rd nearest neighbor S atoms. This indicates that only the reaction activity of the S atoms bonding with impurities are enhanced largely. For the non-mental elements doping ΔG_{H*} on the 1st and 2nd nearest neighbor S atoms show negligible difference.

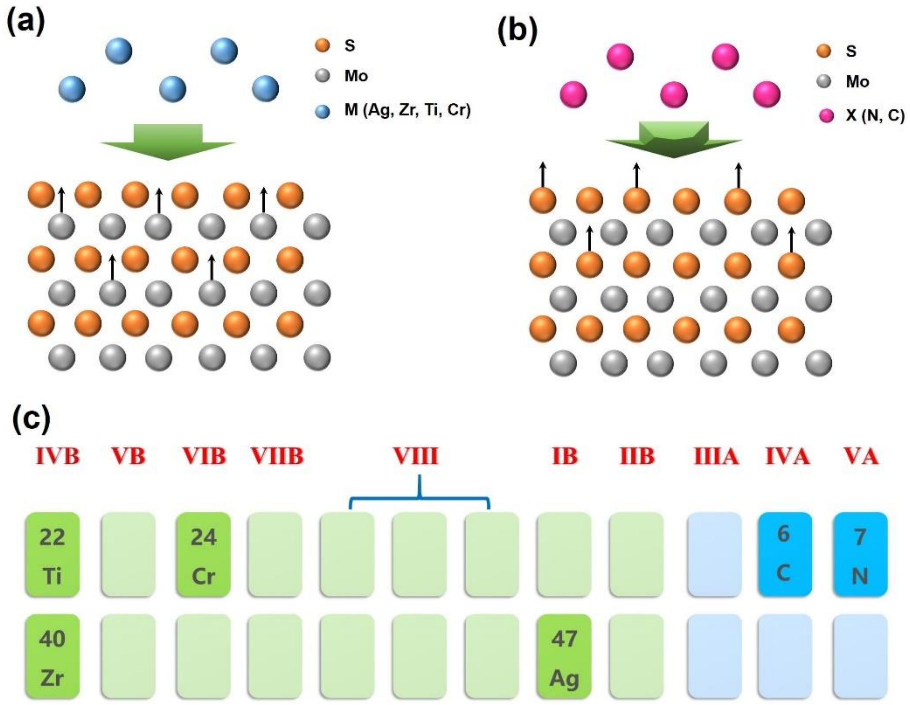

Figure S1. Schematic diagram of plasma injection.

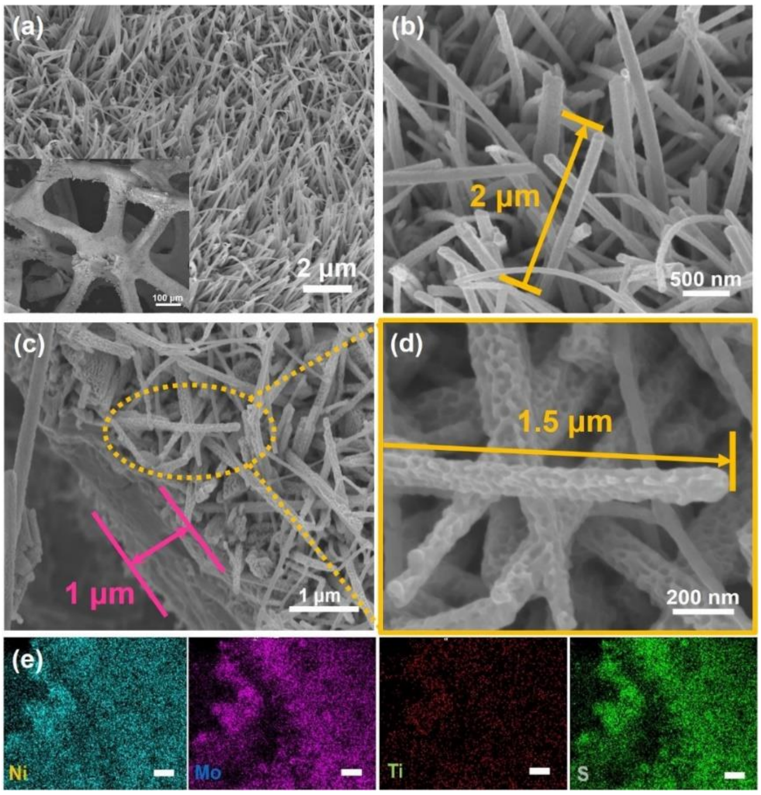

Figure S2. (a-d) SEM images of Ti500-MoS_2@Ni_3S_2/NF, (e) SEM element maps of Ti500-MoS_2@Ni_3S_2/NF.

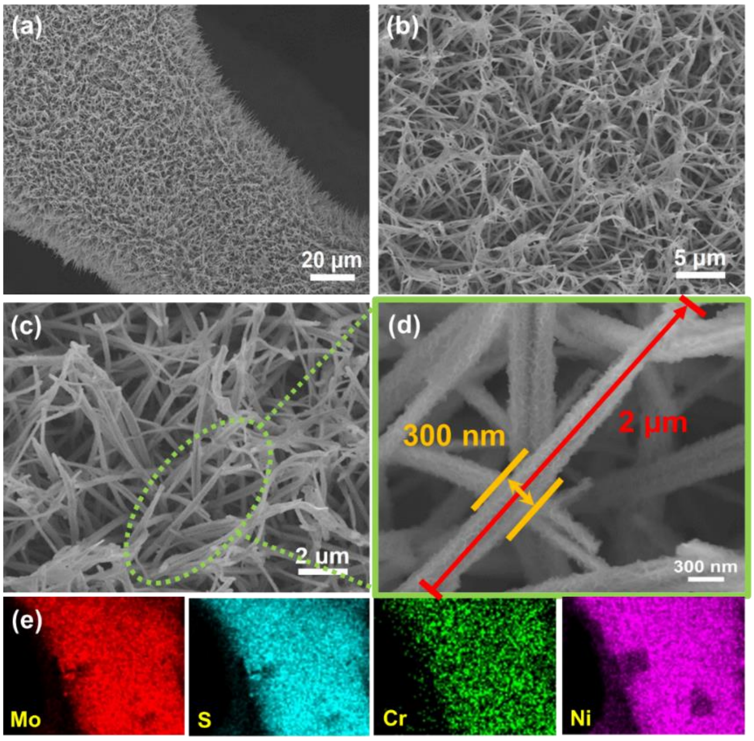

Figure S3. (a-d) SEM images of Cr500-$MoS_2@Ni_3S_2$/NF, (e) SEM element maps of Cr500-$MoS_2@Ni_3S_2$/NF.

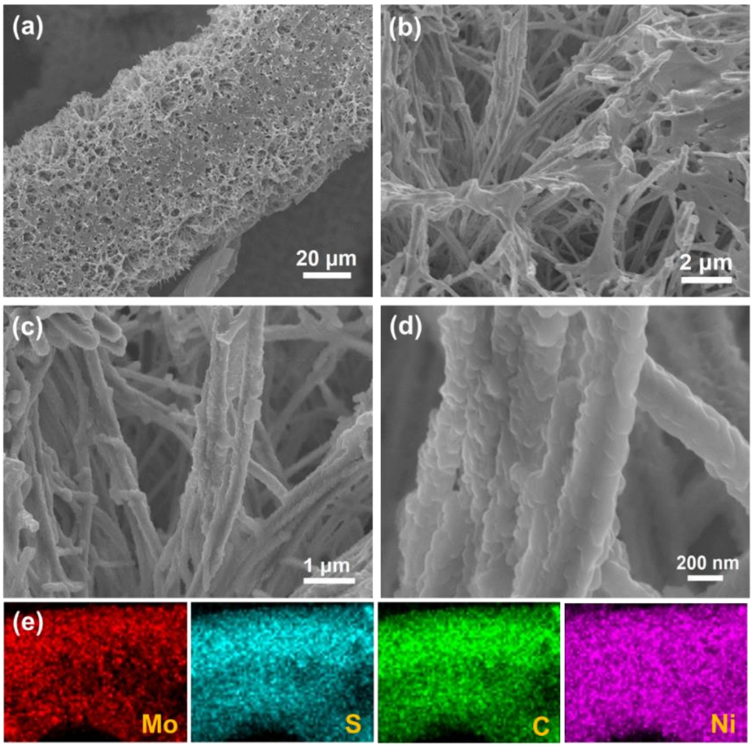

Figure S4. (a-d) SEM images of C1.0-MoS_2@Ni_3S_2/NF, (e) SEM element maps of C1.0-MoS_2@Ni_3S_2/NF.

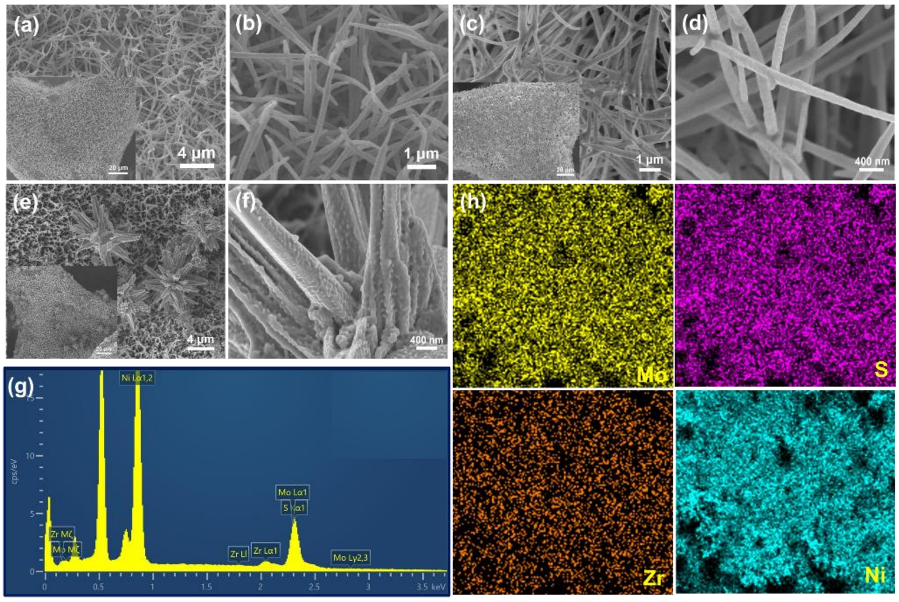

Figure S5. (a, b) SEM images of Zr250-$MoS_2@Ni_3S_2$/NF, (c, d) SEM images of Zr500-$MoS_2@Ni_3S_2$/NF, (e, f) SEM images of Zr1000-$MoS_2@Ni_3S_2$/NF, (g) EDS spectra of Zr500-$MoS_2@Ni_3S_2$/NF, (h) SEM element maps of Zr500-$MoS_2@Ni_3S_2$/NF.

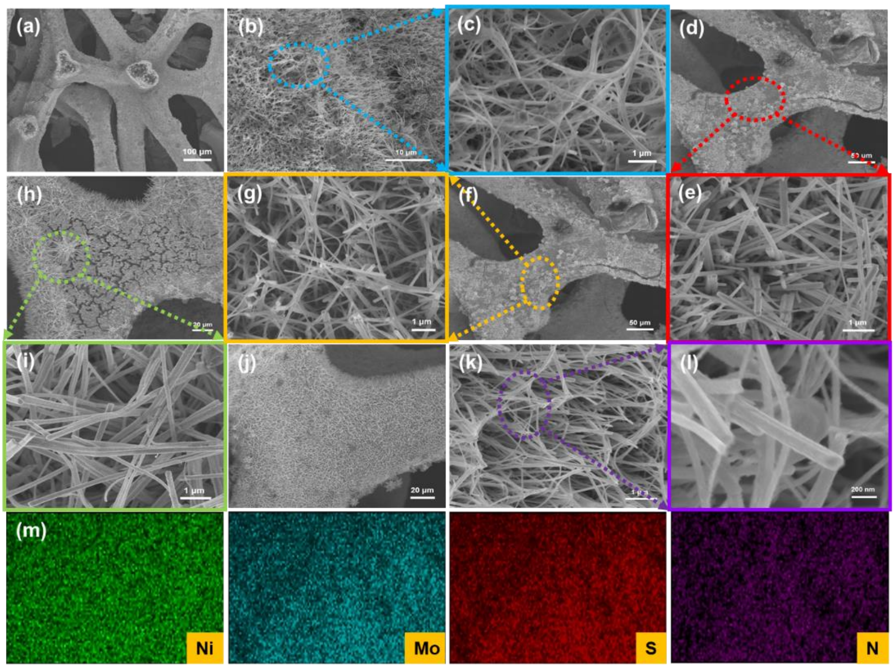

Figure S6. (a-c) SEM images of MoS_2@Ni_3S_2/NF, (d, e) SEM images of N0.5-MoS_2@Ni_3S_2/NF, (f, g) SEM images of N2.0-MoS_2@Ni_3S_2/NF, (h, i) SEM images of N4.0-MoS_2@Ni_3S_2/NF, (j-l) SEM images of N1.0-MoS_2@Ni_3S_2/NF, (m) SEM element maps of N1.0-MoS_2@Ni_3S_2/NF.

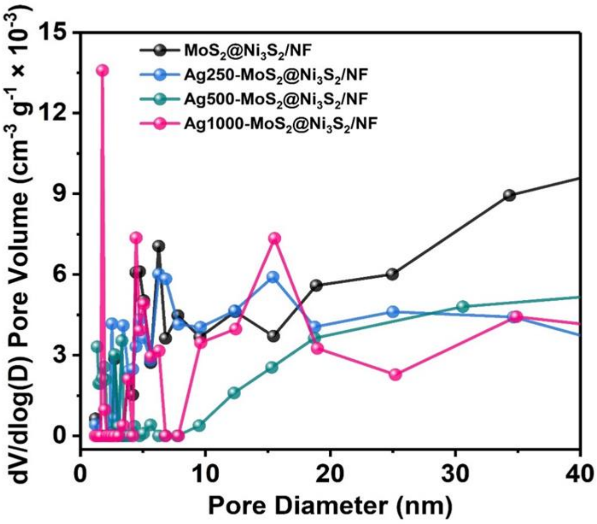

Figure S7. Distribution of pore diameter for the $MoS_2@Ni_3S_2/NF$, Ag250-$MoS_2@Ni_3S_2/NF$, Ag500-$MoS_2@Ni_3S_2/NF$, Ag1000-$MoS_2@Ni_3S_2/NF$.

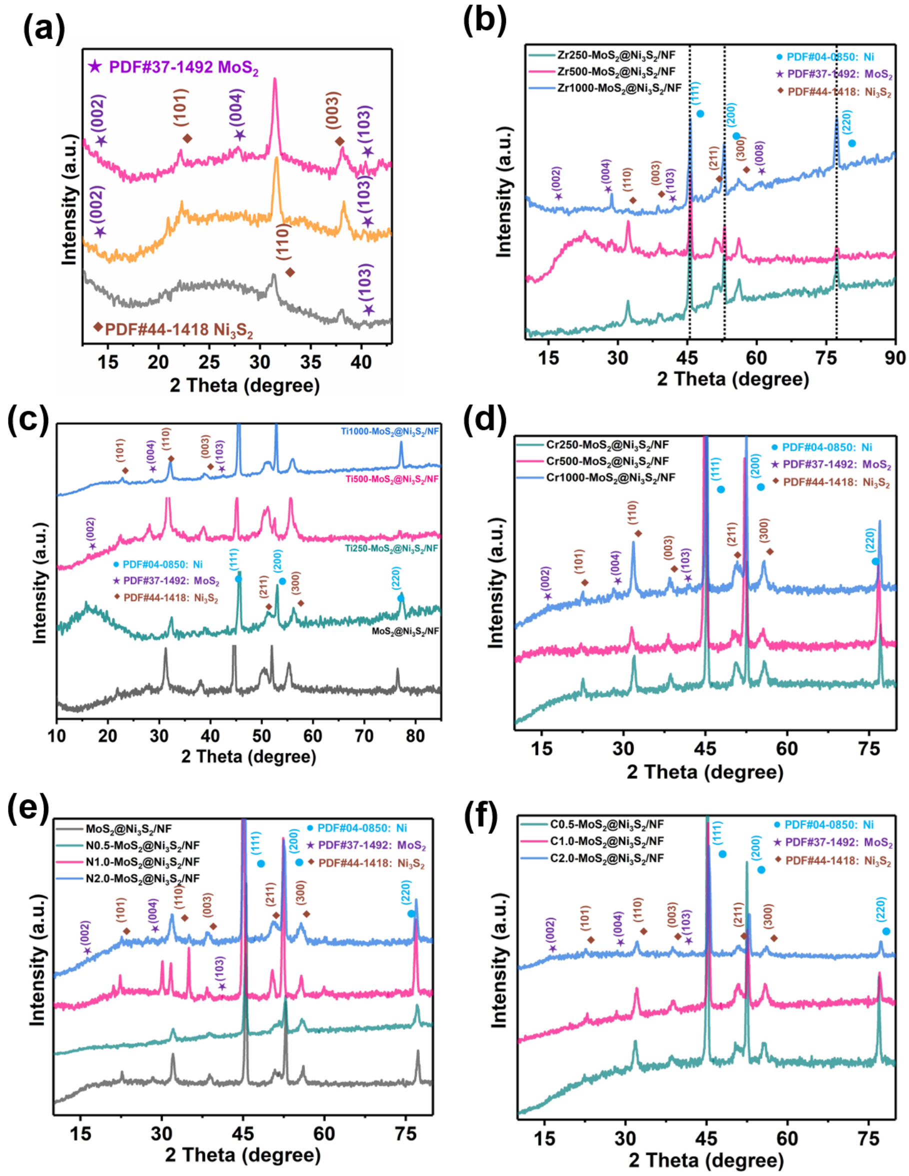

Figure S8. XRD patterns: (a) Ag-doped $MoS_2@Ni_3S_2$/NF. (b) Zr-doped $MoS_2@Ni_3S_2$/NF. (c) Ti-doped $MoS_2@Ni_3S_2$/NF. (d) Cr-doped $MoS_2@Ni_3S_2$/NF. (e) N-doped $MoS_2@Ni_3S_2$/NF. (f) C-doped $MoS_2@Ni_3S_2$/NF.

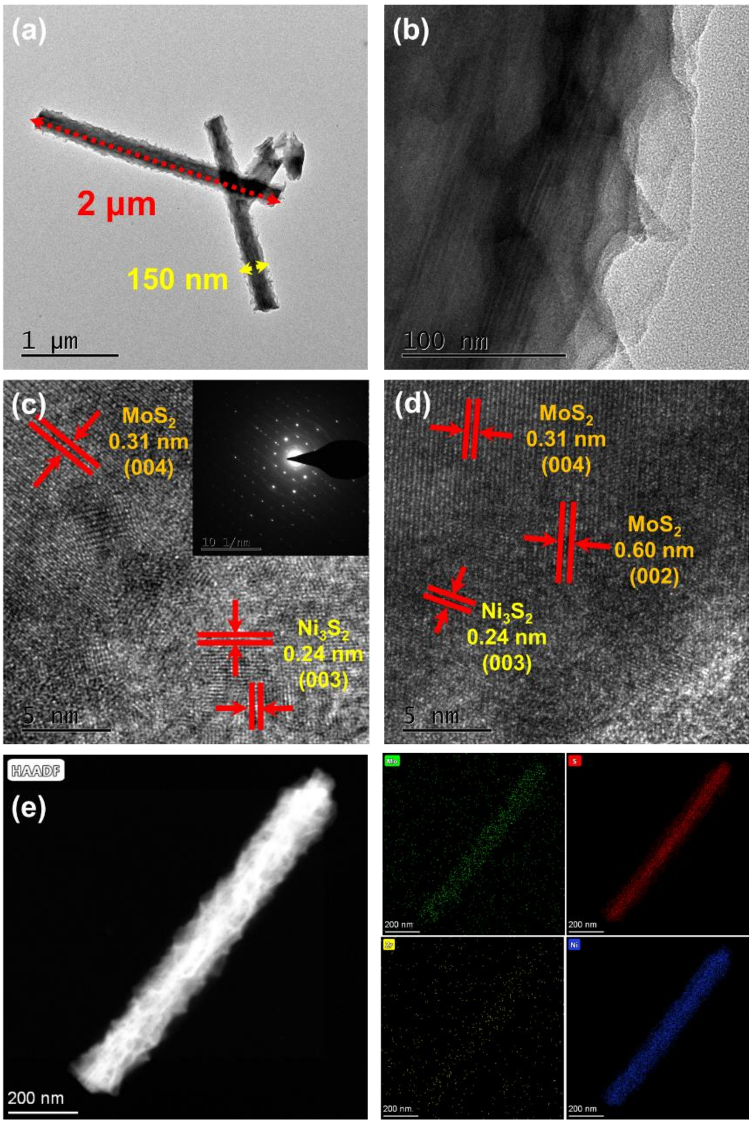

Figure S9. TEM and element maps of Zr500-MoS_2@Ni_3S_2/NF.

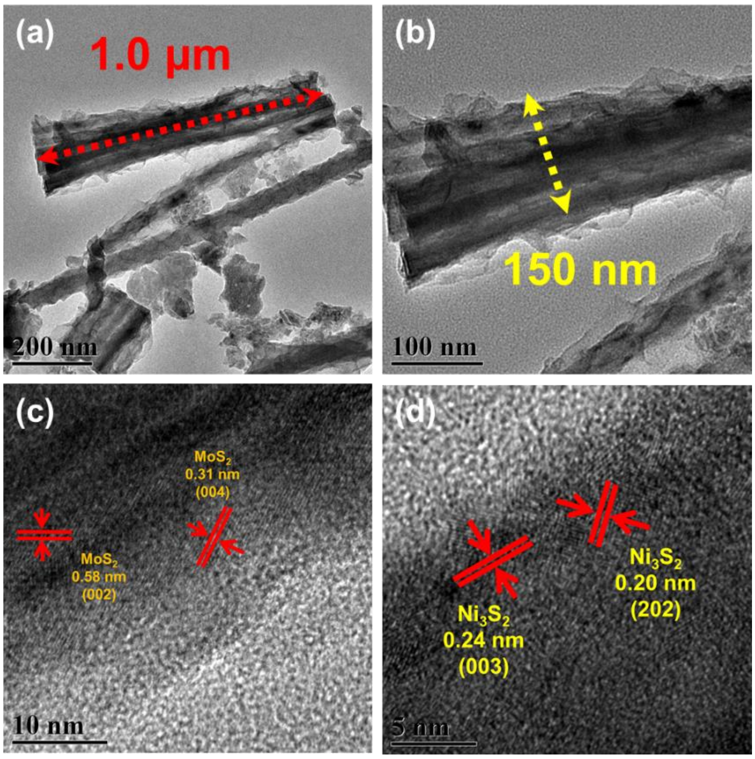

Figure S10. TEM of Ti500-MoS_2@Ni_3S_2/NF.

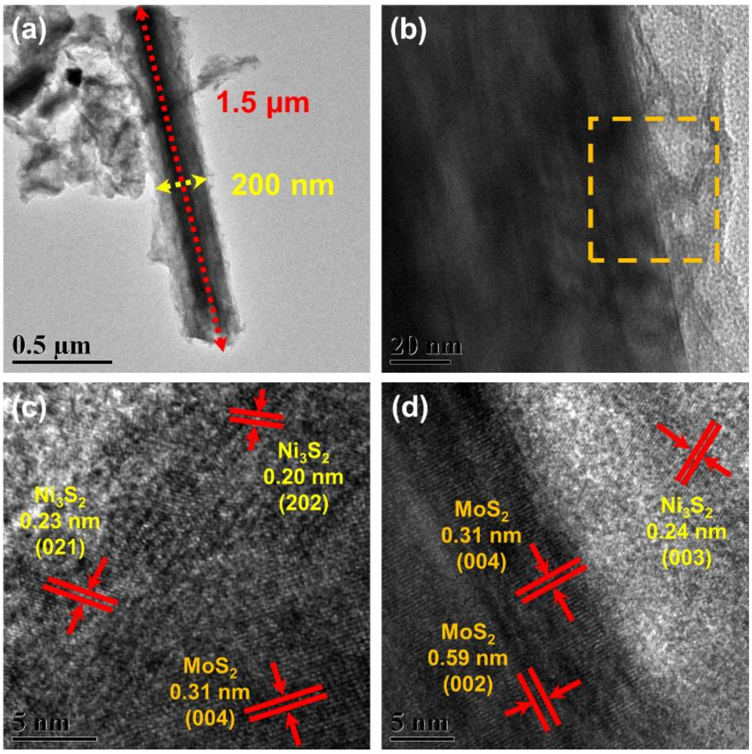

Figure S11. TEM of Cr500-MoS_2@Ni_3S_2/NF.

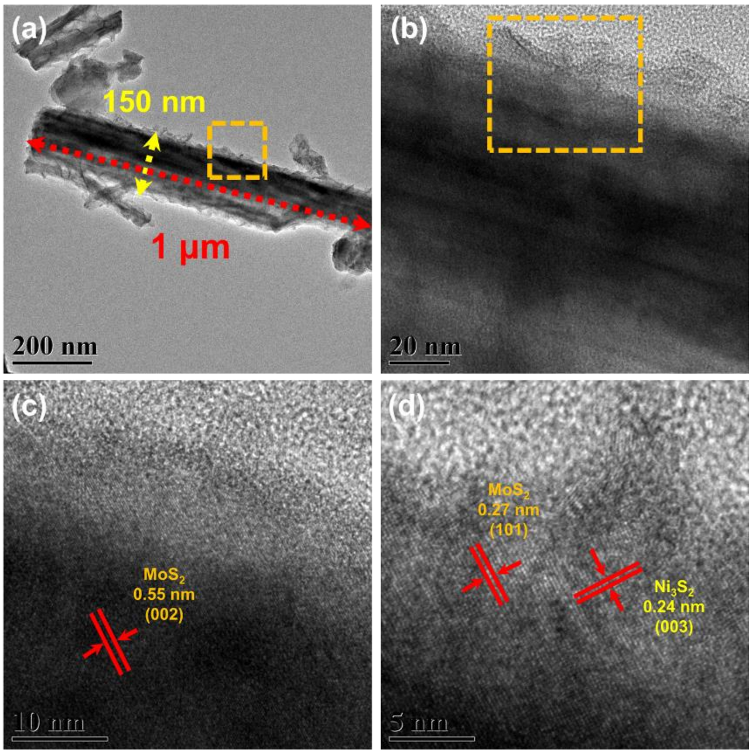

Figure S12. TEM of C1.0-MoS_2@Ni_3S_2/NF.

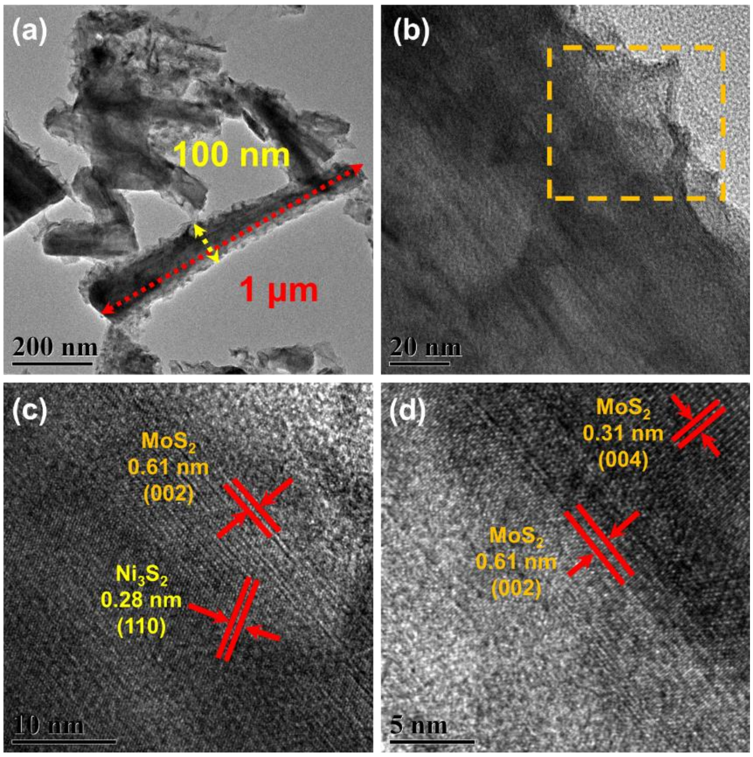

Figure S13. TEM of N1.0-MoS_2@Ni_3S_2/NF.

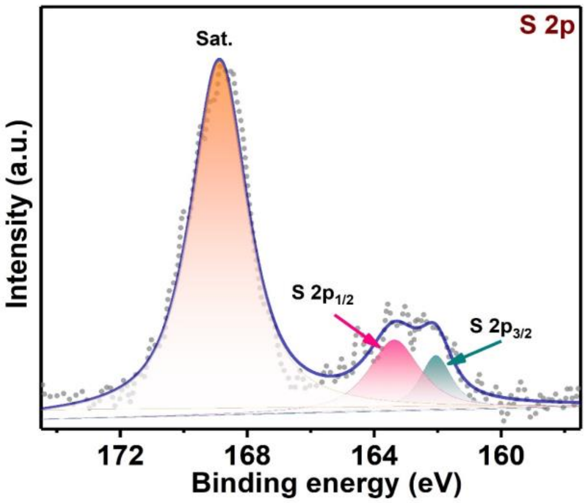

Figure S14. XPS spectra of S 2p for MoS_2@Ni_3S_2/NF.

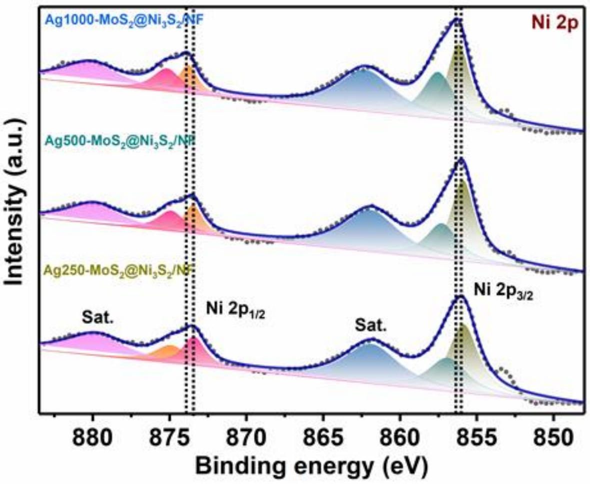

Figure S15. XPS spectra of Ni 2p for Ag-doped $MoS_2@Ni_3S_2$/NF.

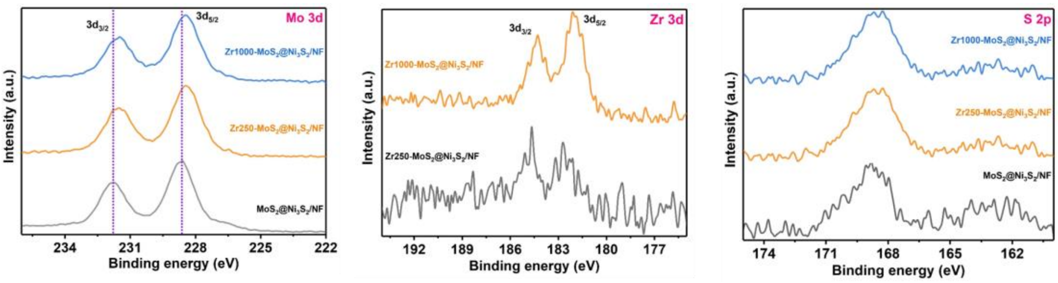

Figure S16. XPS spectra of Zr-doped $MoS_2@Ni_3S_2$/NF.

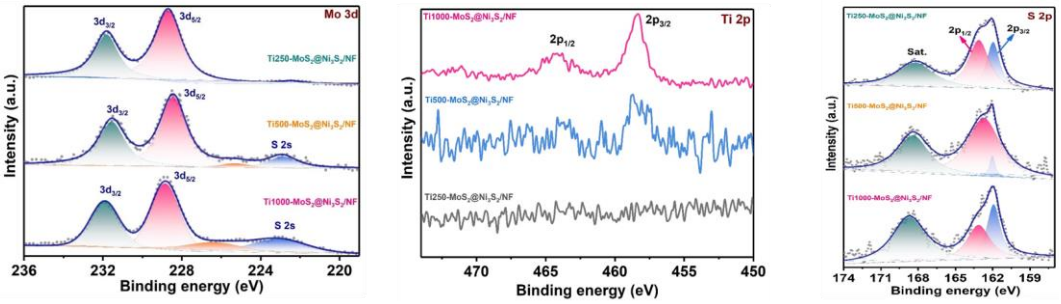

Figure S17. XPS spectra of Ti-doped MoS_2@Ni_3S_2/NF.

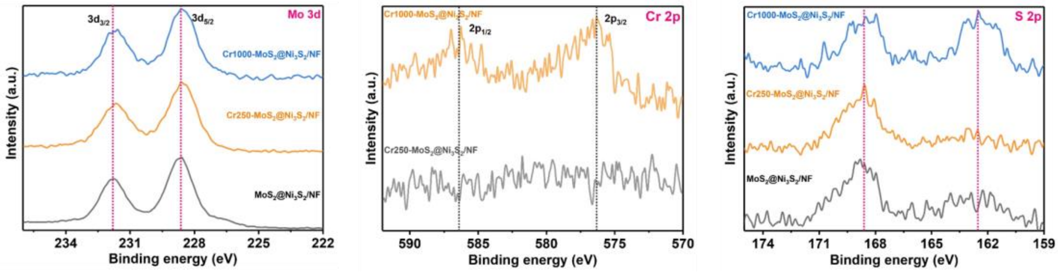

Figure S18. XPS spectra of Cr-doped $MoS_2@Ni_3S_2$/NF.

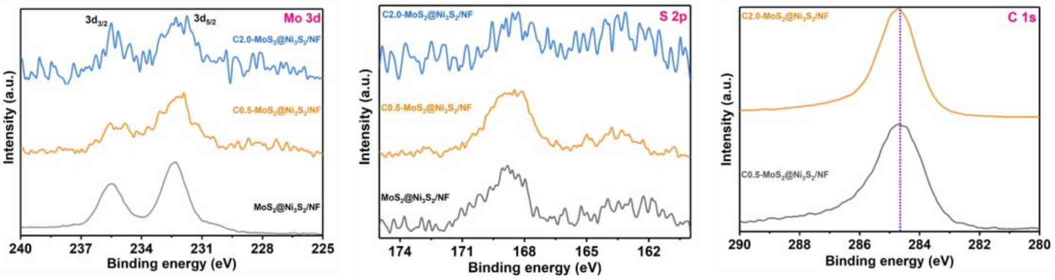

Figure S19. XPS spectra of C-doped $MoS_2@Ni_3S_2/NF$.

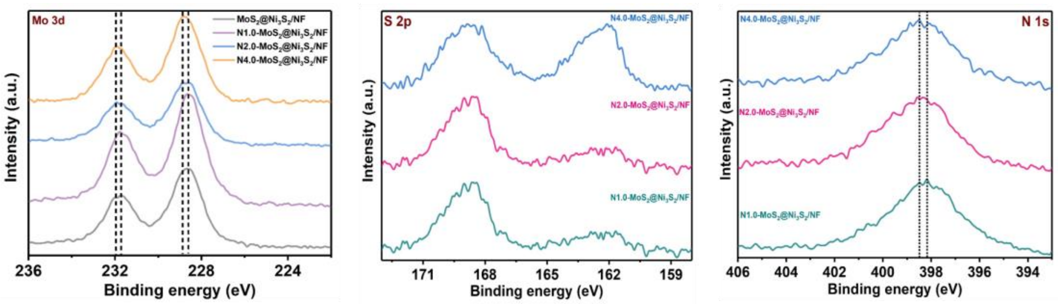

Figure S20. XPS spectra of N-doped $MoS_2@Ni_3S_2$/NF.

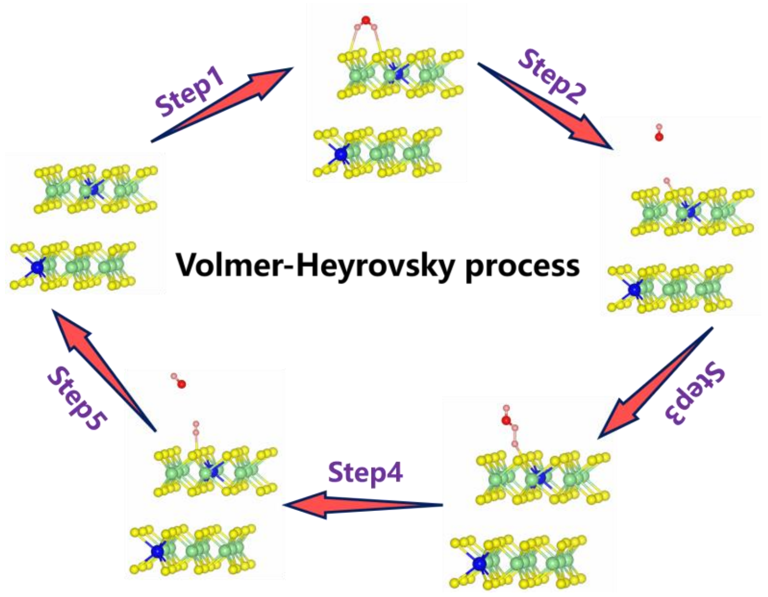

Figure S21. Schematic diagram of the process of hydrogen evolution reaction in an alkaline environment.

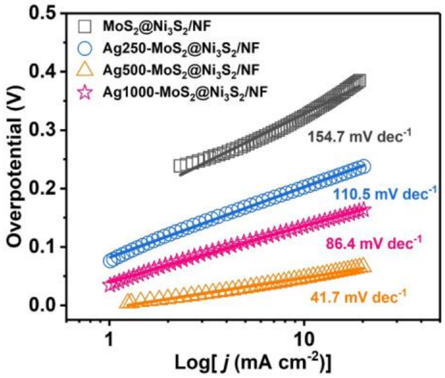

Figure S22. Tafel plots of Ag-doped $MoS_2@Ni_3S_2$/NF.

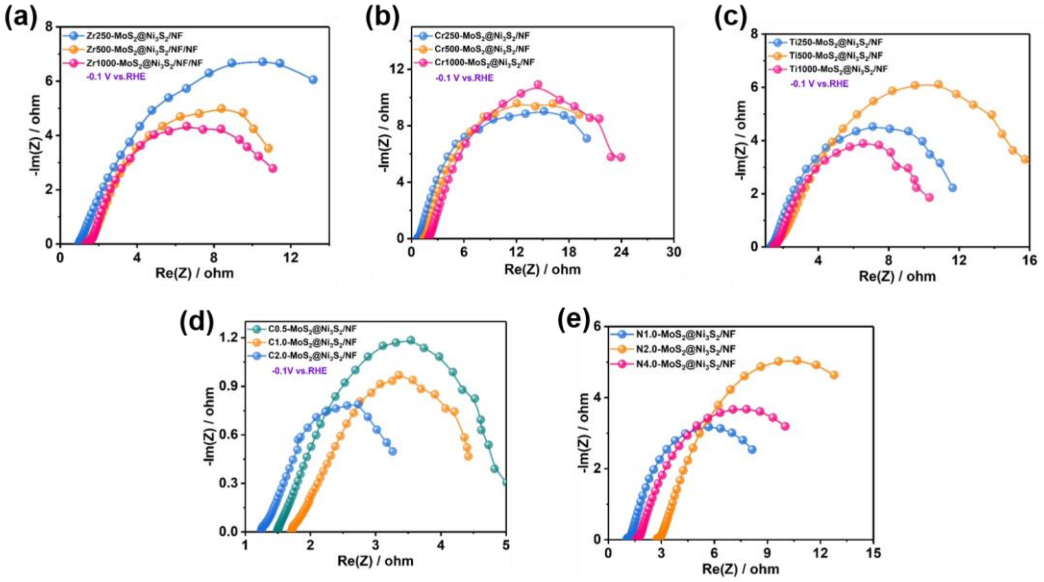

Figure S23. Nyquist plots derived by EIS: (a) Zr-doped $MoS_2@Ni_3S_2$/NF. (b) Cr-doped $MoS_2@Ni_3S_2$/NF. (c) Ti-doped $MoS_2@Ni_3S_2$/NF. (d) C-doped $MoS_2@Ni_3S_2$/NF. (e) N-doped $MoS_2@Ni_3S_2$/NF.

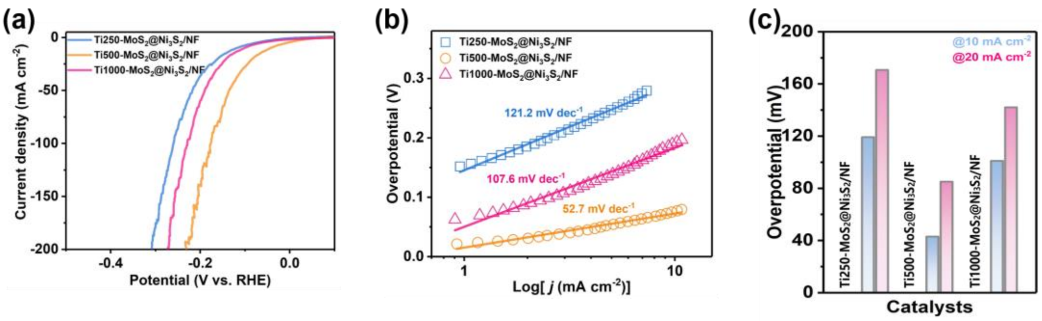

Figure S24. HER characteristics of Ti-doped $MoS_2@Ni_3S_2$/NF: (a) LSV curves, (b) Tafel plots, (c) Overpotentials.

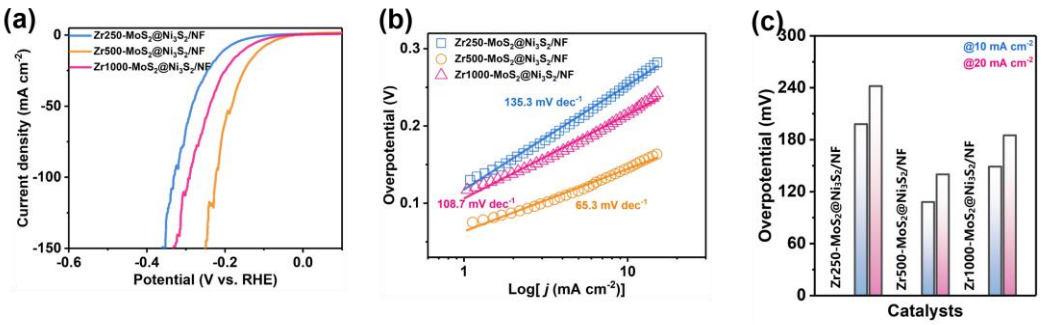

Figure S25. HER characteristics of Zr-doped $MoS_2@Ni_3S_2/NF$: (a) LSV curves, (b) Tafel plots, (c) Overpotentials.

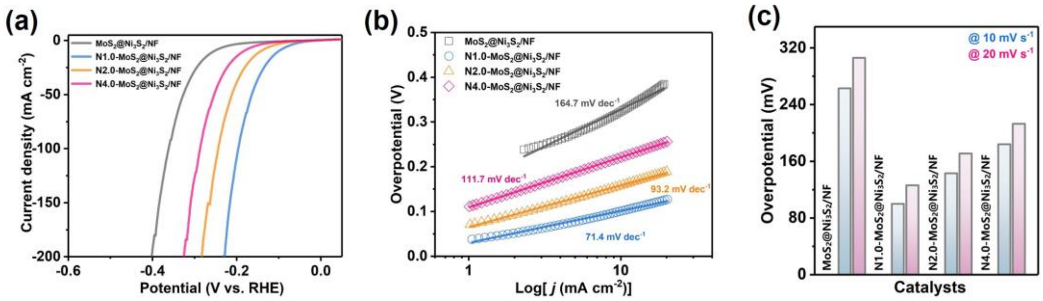

Figure S26. HER characteristics of N-doped $MoS_2@Ni_3S_2/NF$: (a) LSV curves, (b) Tafel plots, (c) Overpotentials.

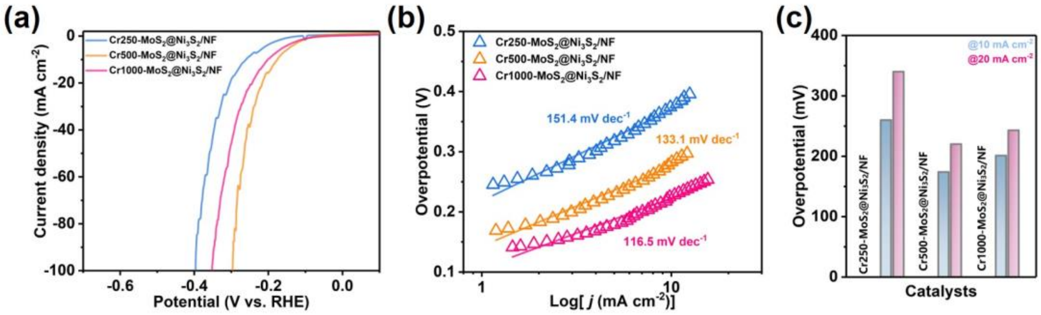

Figure S27. HER characteristics of Cr-doped MoS_2@Ni_3S_2/NF: (a) LSV curves, (b) Tafel plots, (c) Overpotentials.

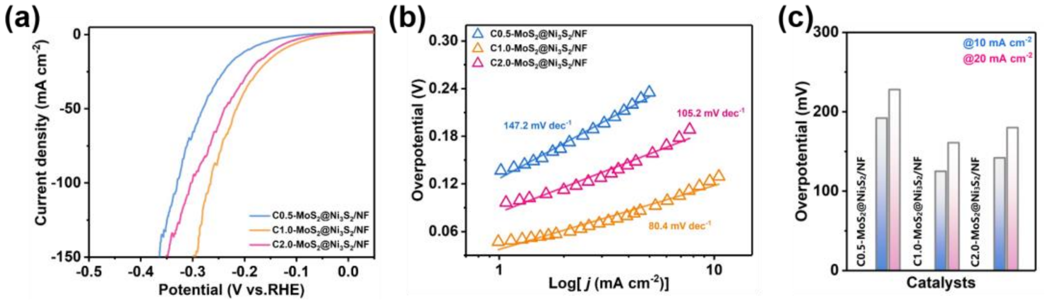

Figure S28. HER characteristics of C-doped $MoS_2@Ni_3S_2$/NF: (d) LSV curves, (e) Tafel plots, (f) Overpotentials.

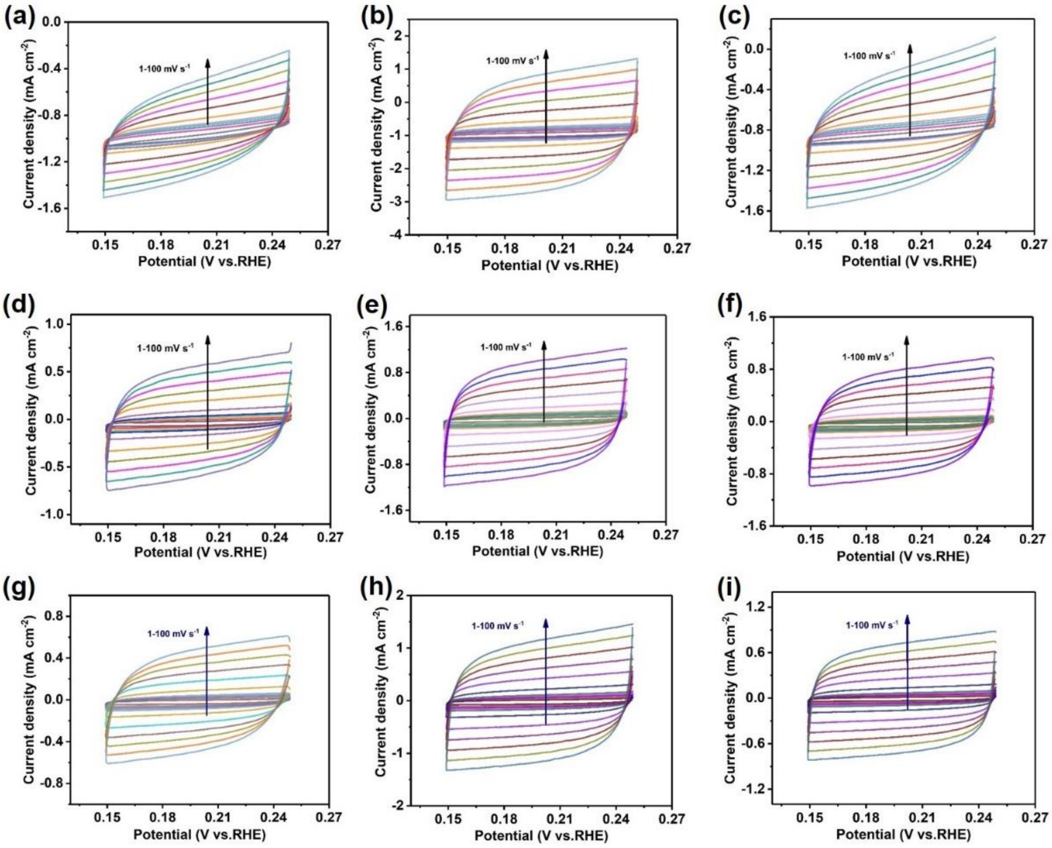

Figure S29. Typical CV curves in 1.0 M KOH with different scan rates: (a-c) Ag-doped MoS_2@Ni_3S_2/NF. (d-f) Ti-doped MoS_2@Ni_3S_2/NF. (g-i) Cr-doped MoS_2@Ni_3S_2/NF.

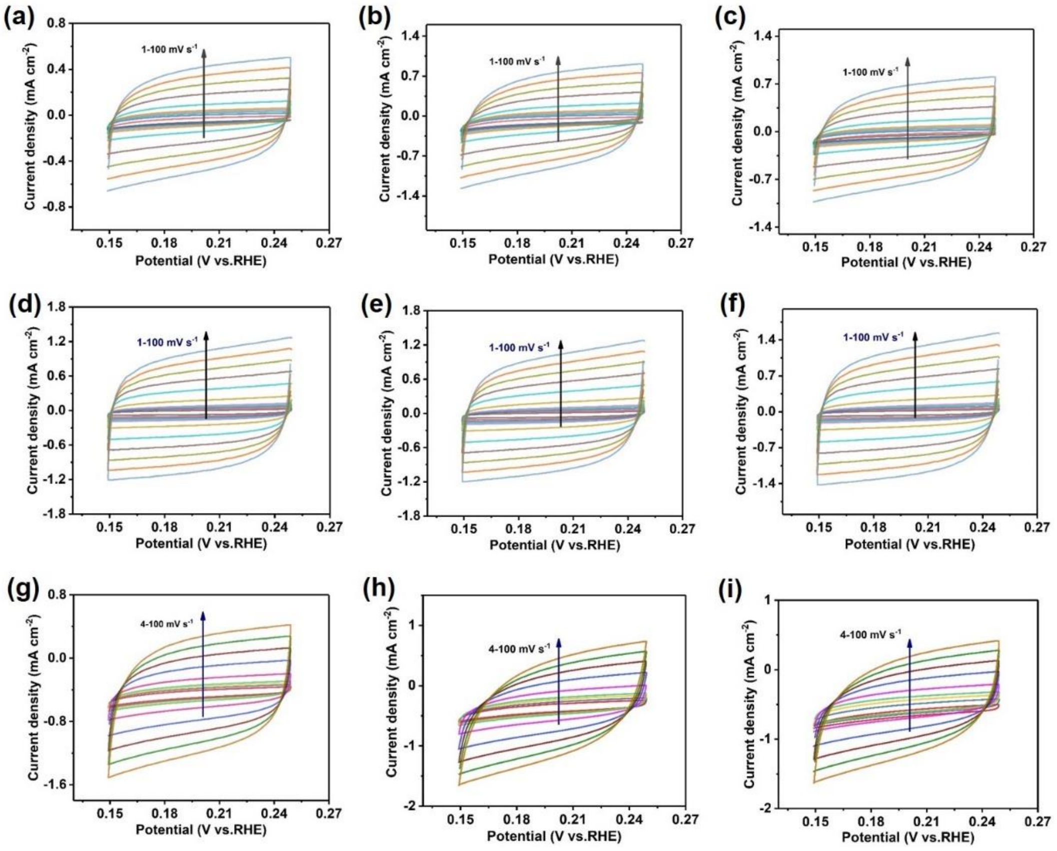

Figure S30. Typical CV curves in 1.0 M KOH with different scan rates: (a-c) Zr-doped $MoS_2@Ni_3S_2$/NF. (d-f) C-doped $MoS_2@Ni_3S_2$/NF. (g-i) N-doped $MoS_2@Ni_3S_2$/NF.

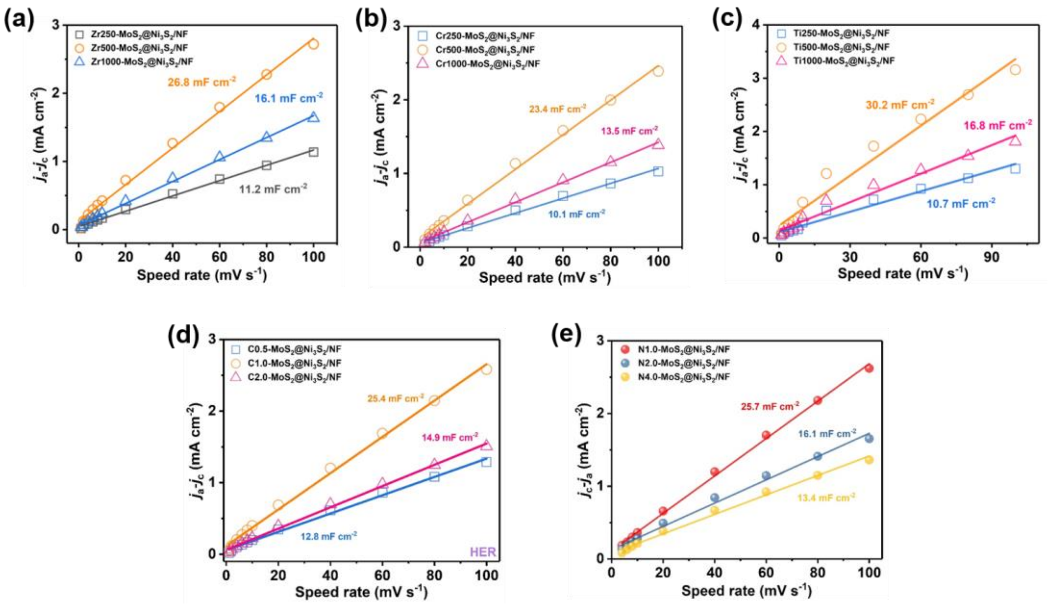

Figure S31. Capacitive current densities at different scanning rates: (a) Zr-doped $MoS_2@Ni_3S_2$/NF. (b) Cr-doped $MoS_2@Ni_3S_2$/NF. (c) Ti-doped $MoS_2@Ni_3S_2$/NF. (d) C-doped $MoS_2@Ni_3S_2$/NF. (e) N-doped $MoS_2@Ni_3S_2$/NF.

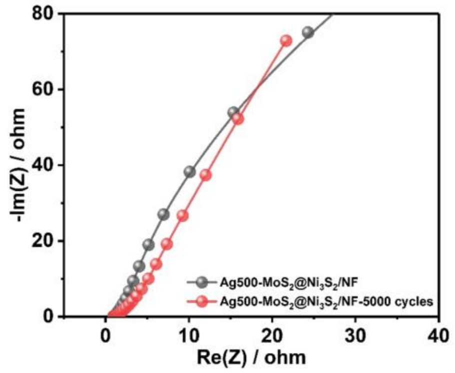

Figure S32. EIS after 5000 cycles.

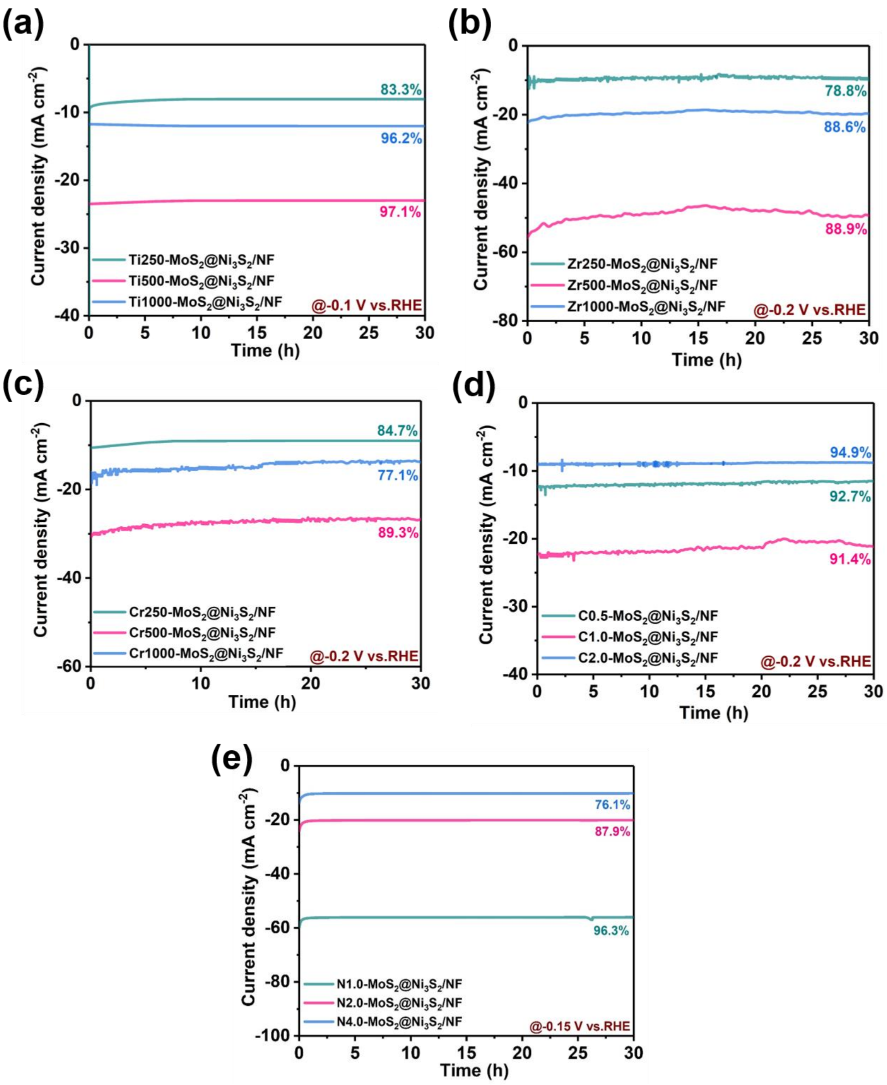

Figure S33. Stability evaluation. (a) Ti-doped $MoS_2@Ni_3S_2$/NF. (b) Zr-doped $MoS_2@Ni_3S_2$/NF. (c) Cr-doped $MoS_2@Ni_3S_2$/NF. (d) C-doped $MoS_2@Ni_3S_2$/NF. (e) N-doped $MoS_2@Ni_3S_2$/NF.

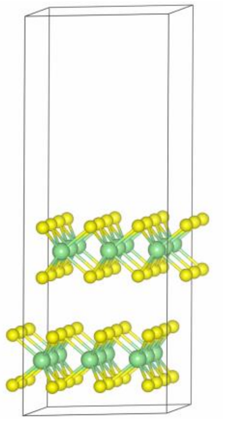

Figure S34. The supercell of MoS_2 surface.

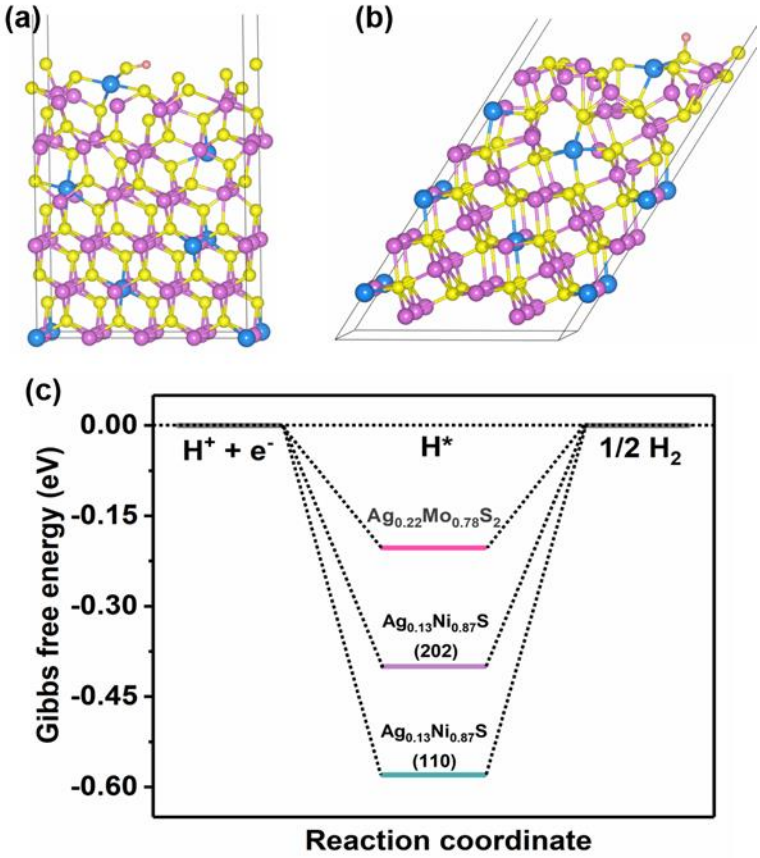

Figure S35. (a) Optimized structure of H^* adsorption on 12.5% Ag-doped NiS (110) surface. (b) Optimized structure of H^* adsorption on 12.5% Ag-doped NiS (202) surface. (c) Gibbs free energies of different surface.

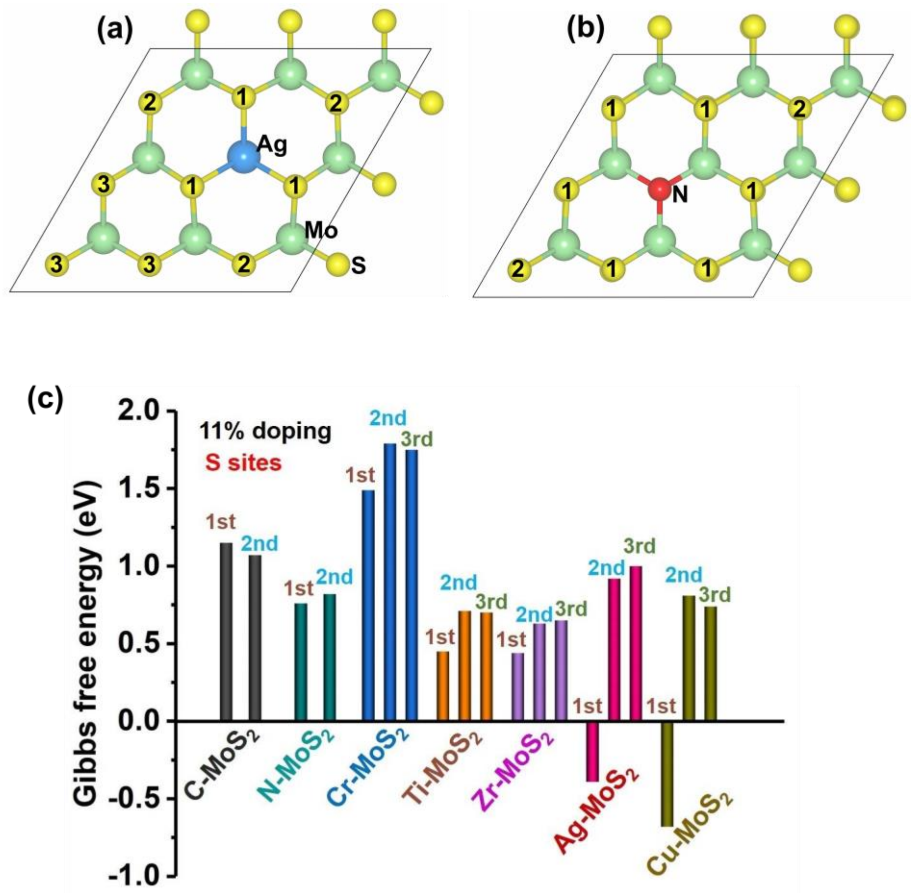

Figure S36. (a) Surface structures of 11 mol% Ag-doped MoS_2 and the 1st, 2nd and 3rd nearest neighbor S atoms of the Ag atom. (b) Surface structures of 11 mol% N-doped MoS_2 and the 1st and 2nd nearest neighbor S atoms of N atom. (e) Gibbs free energies of a H atom adsorption on the 1st, 2nd and 3rd neighbor S atoms of the impurity atom (C, N, Cr, Ti, Zr, Ag, Cu).

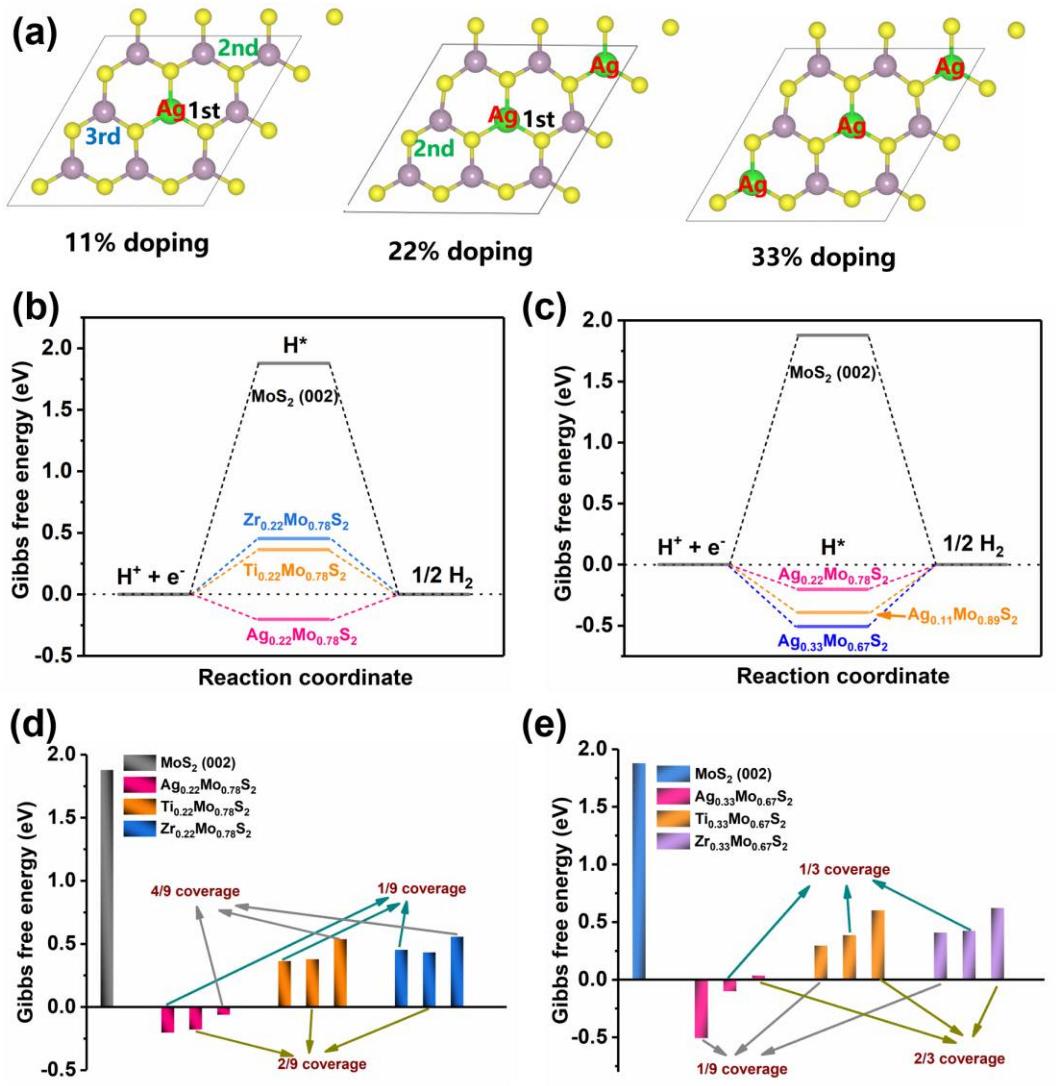

Figure S37. (a) Surface structures of 11, 22, and 33 mol% Ag-doped MoS_2. (b) Gibbs free energies of 22% Ag, Ti, and Zr doped MoS_2. (c) Gibbs free energies of Ag doped MoS_2 with different doping concentrations. (d) Gibbs free energies of 22% Ag, Ti, and Zr doped MoS_2 with different H^* coverage. (e) Gibbs free energies of 33% Ag, Ti, and Zr doped MoS_2 with different H^* coverage.

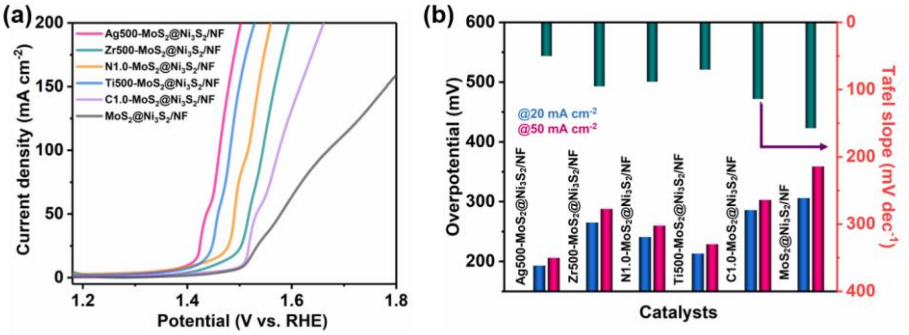

Figure S38. OER characteristics of Ag500-MoS_2@Ni_3S_2/NF, Zr500-MoS_2@Ni_3S_2/NF, N1.0-MoS_2@Ni_3S_2/NF, Ti500-MoS_2@Ni_3S_2/NF, C1.0-MoS_2@Ni_3S_2/NF, and MoS_2@Ni_3S_2/NF: (a) LSV curves, (b) Tafel slopes and overpotentials.

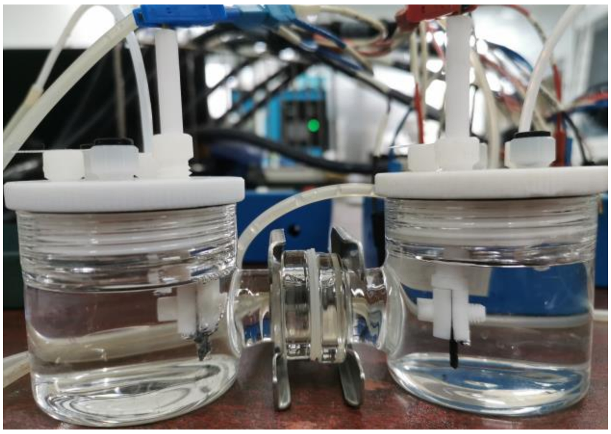

Figure S39. Physical picture of two-electrode water electrolysis process.

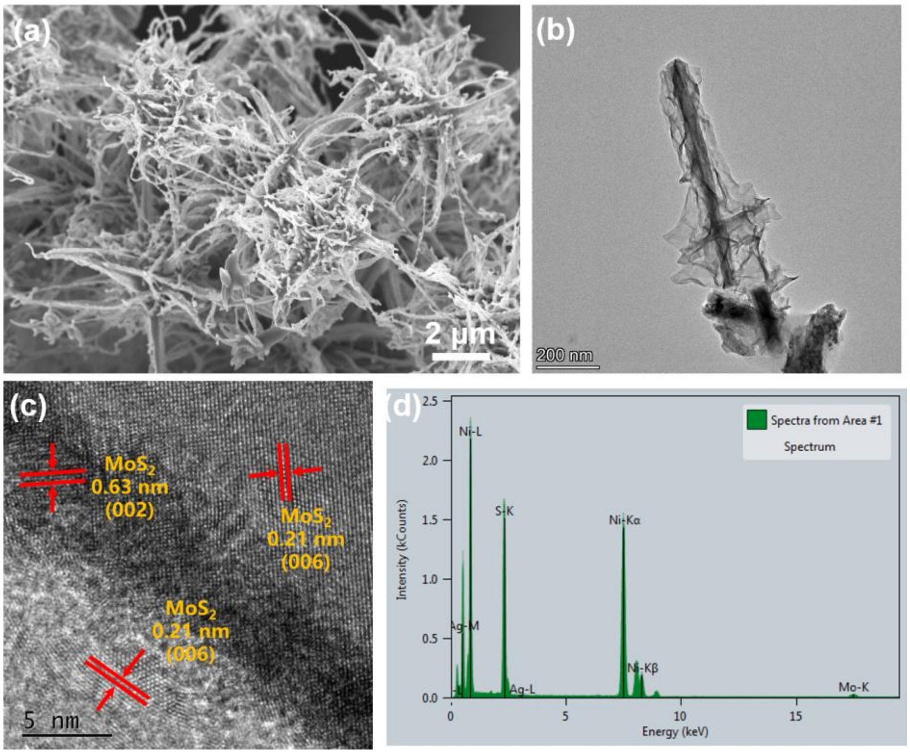

Figure S40. (a) SEM, (b) TEM, (c) HRTEM, (d) EDS of Ag500-MoS_2@Ni_3S_2/NF after stability test.

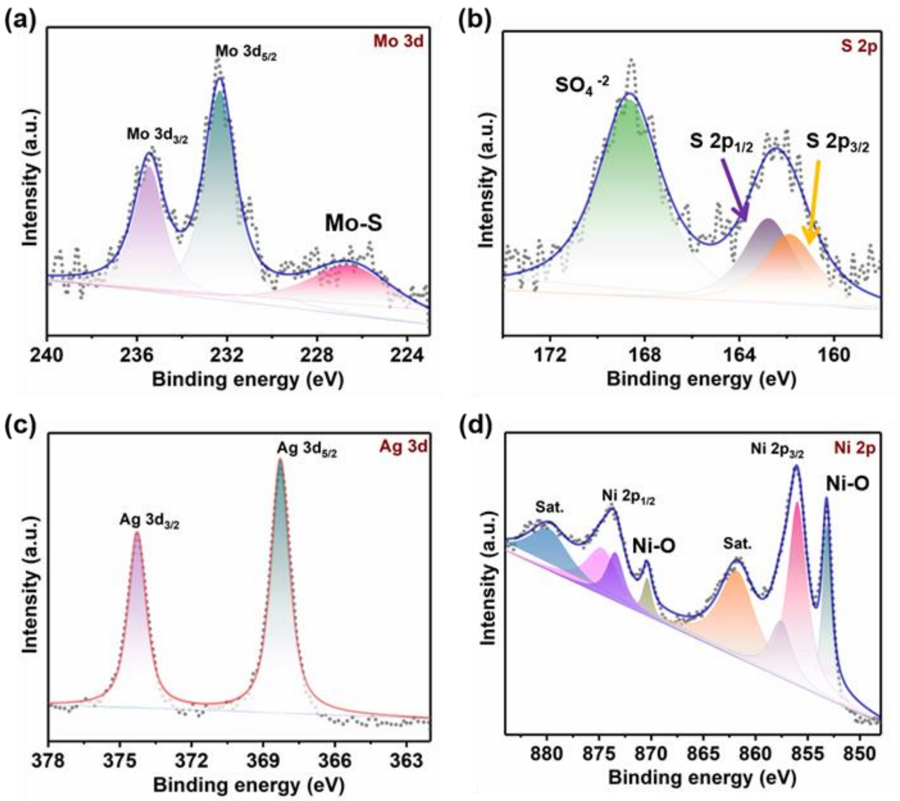

Figure S41. XPS of Ag500-MoS_2@Ni_3S_2/NF after HER stability test (a) Mo 3d, (b) S 2p, (c) Ag 3d, (d) Ni 2p.

Table S1 Ag and Mo relative content ratio.

Catalysts	Ag and Mo content ratio (ICP)	Ag and Mo content ratio (XPS)
Ag250-MoS_2@Ni_3S_2/NF	1.5%	14.4%
Ag500-MoS_2@Ni_3S_2/NF	4.1%	25.1%
Ag1000-MoS_2@Ni_3S_2/NF	8.2%	48.9%

Table S2. Important fitting parameters of the equivalent circuit.

Catalysts	R_s (Ω)	$R_{ct\ s}$ (Ω)	$R_{ct\ dl}$ (Ω)	CPE_s-T	CPE_s-P	CPE_{dl}-T	CPE_{dl}-P
MoS_2@Ni_3S_2/NF	1.02	82.68	4.99	0.057	0.879	1.034	0.157
Ag250-MoS_2@Ni_3S_2/NF	1.20	2.25	62.47	0.371	0.365	0.039	0.723
Ag500-MoS_2@Ni_3S_2/NF	0.91	1.35	0.33	0.039	0.852	0.088	0.555
Ag1000-MoS_2@Ni_3S_2/NF	1.20	45.35	0.146	0.031	0.786	0.054	0.647

References

[S1] G. Kresse, J. Furthmüller, *Phys. Rev. B* **1996**, 54, 11169.

[S2] J. P. Perdew, K. Burke, M. Ernzerhof, *Phys. Rev. Lett*. **1996**, 77, 3865.

[S3] P. E. Blöchl, *Phys. Rev. B* **1994**, 50, 17953.

[S4] S. L. Dudarev, G. A. Botton, S. Y. Savrasov, C. J. Humphreys, A. P. Sutton, *Phys. Rev. B* **1998**, 57, 1505.
